# Microbiome of rehydrated corn and sorghum grain silages treated with microbial inoculants in different fermentation periods

**DOI:** 10.1038/s41598-022-21461-4

**Published:** 2022-10-07

**Authors:** Mariele Cristina Nascimento Agarussi, Odilon Gomes Pereira, Felipe Evangelista Pimentel, Camila Ferreira Azevedo, Vanessa Paula da Silva, Fabyano Fonseca e Silva

**Affiliations:** 1 Department of Animal Science, Federal University of Vicosa, Avenida PH. Rolfs, Vicosa, Mina Gerais 36570-900 Brazil; 2 Departament of Statistics, Federal University of Vicosa, Avenida PH. Rolfs, Vicosa, 36570-900 Brazil

**Keywords:** Microbial communities, Plant sciences

## Abstract

Due to the co-evolved intricate relationships and mutual influence between changes in the microbiome and silage fermentation quality, we explored the effects of *Lactobacillus plantarum* and *Propionibacterium acidipropionici* (Inoc1) or *Lactobacillus buchneri* (Inoc2) inoculants on the diversity and bacterial and fungal community succession of rehydrated corn (CG) and sorghum (SG) grains and their silages using Illumina Miseq sequencing after 0, 3, 7, 21, 90, and 360 days of fermentation. The effects of inoculants on bacterial and fungal succession differed among the grains. *Lactobacillus* and *Weissella* species were the main bacteria involved in the fermentation of rehydrated corn and sorghum grain silage. *Aspergillu*s spp. mold was predominant in rehydrated CG fermentation, while the yeast *Wickerhamomyces anomalus* was the major fungus in rehydrated SG silages. The Inoc1 was more efficient than CTRL and Inoc2 in promoting the sharp growth of *Lactobacillus* spp. and maintaining the stability of the bacterial community during long periods of storage in both grain silages. However, the bacterial and fungal communities of rehydrated corn and sorghum grain silages did not remain stable after 360 days of storage.

## Introduction

Corn and sorghum grains have been used in concentrates offered to ruminants to provide energy mainly from their starch content^1^. The grain endosperm contains the highest amount of starch and determines the economic and nutritional value of the grain because the structure and composition of starch and its physical interaction with grain protein can alter its digestibility^2^. The effect of the endosperm on digestibility can be manipulated by grain processing^3^. Rehydrated grain silage is a promising technique to improve the nutritive value of grains^4^, and among grains, sorghum has the highest gain in digestibility after this process, followed by corn grain and other cereals^5^.

During the ensiling process, the increase in grain starch digestibility may be due to partial degradation of the hydrophobic starch-protein matrix that surrounds the starch granules by proteolysis^6^, resulting in greater prolamin solubilization and increased starch granule surface area for potential attack by rumen bacteria^7^.

Studies have shown that climatic conditions affect all stages of silage production and utilization, especially in hot and humid areas, because microbial proliferation is strongly influenced by temperature^8^. These climatic factors not only affect forage crop growth and disease incidence but also influence silage fermentation and aerobic stability^9^.

*Lactobacillus plantarum* has been reported to be the most commonly used silage inoculant^10^. This species produces lactic acid, which rapidly reduces pH and improves fermentation^11^. However, high amounts of silage have been lost, and the cost of production may suffer negative consequences due to aerobic deterioration; therefore, propionic bacteria and heterofermentative bacteria producing acetate have been studied to reduce the deterioration of silages after exposure to air^4,12^.

Bacterial inoculation can influence the fermentation characteristics and silage nutritional value differently depending on the epiphytic bacteria present in the raw material^13^ and the strains in different silage materials^14^. According to Si et al*.*^13^, silage and its microbiota have co-evolved intricate relationships, and mutual influence exists between changes in the microbiome and silage fermentation parameters, such as the positive correlation between *Lactobacillus plantarum* and lactic acid content. Generally, the composition of microorganisms before and after ensiling undergoes significant changes^15^. Monitoring these changes during ensiling would be helpful for thoroughly understanding and improving the ensiling process^16^.

Recently, molecular methods have been used to evaluate the quality, activity, and dynamics of a complex community of microorganisms involved in forage preservation^17–19^, avoiding the underestimation of microbial diversity of silages^20^. Despite the potential of rehydrated grain silages and the fact that silage-associated microorganisms may significantly affect silage quality and ruminant health, few studies have evaluated the microbial population and its dynamics during the fermentation process of rehydrated corn and sorghum grains, particularly using next-generation sequencing.

In this context, expecting a rapid change in the silage environment and different effects of microbial inoculants on the microbiome of silages, we explored the succession of bacterial and fungal populations, identified the dominant microorganisms involved in ensiling, and evaluated the impact of microbial inoculants on the epiphytic community of rehydrated corn and sorghum grains and their silages after 0, 3, 7, 21, 90, and 360 days of fermentation.

## Methods

### Location and climatic conditions

The experiment was conducted between January 2016 and January 2017 at the Department of Animal Science of the Federal University of Vicosa (Viçosa, MG, Brazil), located 20° 45′ S, 42° 52′ W, 663 m above sea level. The annual precipitation and average temperature during the year of the experiment were 1235.4 mm and 20.7 °C, respectively.

### Ensiling and sampling

The samples used in this experiment were obtained from a previous study conducted by^21^ (unpublished data), who evaluated the effects of microbial inoculant and fermentation period length on rehydrated corn and sorghum grain silages. The current study complies with Brazilian ethical regulations. All methods were performed in accordance with relevant guidelines and regulations. Briefly, the experiment was carried out under a completely randomized design (with three replicates) based on a 2 × 3 × 6 factorial assay, with two grains (corn-CG and sorghum-SG), three inoculants, and six fermentation periods (0, 3, 7, 21, 90, and 360 days). The evaluated treatments were corn control (CG-CTRL), corn Lalsil Milho (CG-Inoc1), corn Lalsil AS (CG-Inoc2), sorghum control (SG-CTRL), sorghum Lalsil Milho (SG-Inoc1), and sorghum Lalsil AS (SG-Inoc2). The inoculants were composed of CTRL—non-inoculated; Inoc1—*Lactobacillus plantarum* > 3.0 × 10^10^ CFU g^−1^*, Propionibacterium acidipropionici* > 3.0 × 10^10^ CFU g^−1^, and sucrose (Lalsil Milho, Lallemand Animal Nutrition); Inoc2—*Lactobacillus buchneri* CNCM-I 4323 1.0 × 10^11^ CFU g^−1^ and sucrose (Lalsil AS, Lallemand Animal Nutrition) at an application rate of 10^5^ CFU g^−1^.

Corn and sorghum grains from a local farm were grossly disintegrated in a mill retrofitted with 3 mm mesh sieves. Prior to fermentation, the milled grains were rehydrated (with water) to a moisture content of 30%. Subsequently, inoculants were dissolved in distilled water at the dosage recommended by the manufacturer, sprayed on 500 g of rehydrated grains, mixed uniformly by hand, packed into plastic film bags (25.4 cm × 35.56 cm), and vacuumed with a vacuum sealer (Eco vacuum 1040, Orved, Italy). The same amount of water was applied to CTRL silages.

The bags were stored in the laboratory at room temperature (range 23–27 °C). From the total of 108 bags, 18 were opened 0, 3, 7, 21, 90 and 360 days after fermentation. Representative composite samples of each treatment in each fermentation period were prepared, totalizing six samples per fermentation period and 36 total samples.

### DNA extraction and sequencing

The 36 samples were crushed in liquid nitrogen, and total DNA was extracted using the NucleoSpin Soil DNA extraction kit (Macherey–Nagel, Düren, Germany), according to the manufacturer’s recommendations. The DNA was quantified using a Nanodrop spectrophotometer (Thermo Scientific, Waltham, USA) and checked for quality on 1.4% agarose gel.

Briefly, PCR amplicon libraries targeting the 16S rRNA-encoding gene present in metagenomic DNA were produced using a barcoded primer set adapted for Illumina HiSeq2000 and MiSeq^22^. Subsequently, DNA sequence data were generated using Illumina paired-end sequencing at the Environmental Sample Preparation and Sequencing Facility (ESPSF) at Argonne National Laboratory, USA. Specifically, the V4 region of the 16S rRNA gene (515F-806R) was PCR-amplified with region-specific primers that included sequencer adapter sequences used in the Illumina flow cell^22,23^.

The reaction was supplemented with a custom peptide nucleic acid (PNA) blocker designed to prevent the amplification of contaminating host sequences (mitochondrial or plastid). These blockers clamp onto host sequences during the PCR process and prevent their amplification^24^. The reverse amplification primer also contained a twelve-base barcode sequence that supported the pooling of up to 2167 different samples in each lane^22,23^.

Each 25 µL PCR reaction contained 8.5 µL of Mo BIO PCR Water (certified DNA-free), 12.5 µL of QuantaBio’s AccuStart II ToughMix (2 × concentration, 1 × final), 1 µL Golay barcode tagged reverse primer (5 µM concentration, 200 pM final), 1 µL forward primer (5 µM concentration, 200 pM final), 1 µL PNA blocker (mitochondrial or plastid, 25 µM concentration, 1 µM final) (Lundberg et al., 2013), and 1 µL of template DNA. The conditions for PCR were as follows (Lundberg et al., 2013): 95 °C for 45 s to denature the DNA, followed by 35 cycles at 95 °C for 15 s, 78 °C for 10 s (for PNA annealing), 50 °C for 30 s (for primer annealing), and 72 °C for 30 s, with a cooldown to 4 °C once the cycling was completed. The amplicons were quantified using PicoGreen (Invitrogen, Waltham, USA) and a plate reader (Infinite 200 PRO, Tecan). Once quantified, the volumes of each product were pooled into a single tube so that each amplicon was represented in equimolar amounts. This pool was then cleaned using AMPure XP Beads (Beckman Coulter) and quantified using a fluorometer (Qubit, Invitrogen).

After quantification, the molarity of the pool was determined, diluted down to 2 nM, denatured, and then diluted to a final concentration of 6.75 pM with a 10% PhiX spike for sequencing with Illumina MiSeq. Amplicons were sequenced on a 251 bp × 12 bp × 251 bp MiSeq run using customized sequencing primers and procedures^22^.

For fungal analyses, the genomic DNA was amplified using a custom-barcoded internal transcribed spacer (ITS) primer set adapted for Illumina HiSeq2000 and MiSeq. These primers were designed by Kabir Peay’s laboratory at Stanford University (Plos one; Smith, Peay 2014). The reverse amplification primer also contained a twelve-base barcode sequence that supported the pooling of up to 2167 different samples in each lane^22,25^. Each 25 µL PCR reaction contained 12 µL of Mo BIO PCR Water (certified DNA-free), 10 µL of 5 Prime HotMasterMix (1 ×), 1 µL of forward primer (5 µM concentration, 200 pM final), 1 µL Golay barcode tagged reverse primer (5 µM concentration, 200 pM final), and 1 µL of template DNA. The conditions for PCR were as follows: 94 °C for 3 min to denature the DNA, followed by 35 cycles at 94 °C for 45 s, 50 °C for 60 s, and 72 °C for 90 s, with a final extension of 10 min at 72 °C to ensure complete amplification. The amplicons were quantified using PicoGreen (Invitrogen) and a plate reader.

Once quantified, different volumes of each product were pooled into a single tube so that each amplicon was equally represented. This pool was then cleaned using the UltraClean PCR Clean-Up Kit (Mo BIO, Carlsbad, USA) and quantified using a Qubit (Invitrogen). After quantification, the molarity of the pool was determined, diluted down to 2 nM, denatured, and then diluted to a final concentration of 6.75 pM with a 10% PhiX spike for sequencing with Illumina MiSeq.

### Bioinformatics analyzes

Raw sequencing reads obtained from 16S rRNA and ITS rRNA amplicon sequencing were subjected to different quality-filtering steps. Sequences that showed bases with a maximum expected error of 0.05 probability were removed, and the remaining sequences were grouped into operational taxonomic units (OTUs) using the program Usearch v.11^26^, with a threshold of 97% similarity. Chimeras were removed using the Uparse algorithm^27^. The ITSx v.1.0.11 program^28^ was used to remove non-fungal ITS1 sequences.

Taxonomic annotation was performed using the Basic Local Alignment Search Tool (BLAST) of QIIME v.1.9.1^25^, using the SILVA and UNITE databases for bacterial and fungal populations, respectively. Contaminant sequences such as those of chloroplast and mitochondria were removed through taxonomic annotation. Alpha diversity metrics (Chao1 richness, evenness, and Simpson diversity) were calculated using the R software (Version 2.15.3).

### Statistical model

The normal, Poisson, inflated Poisson, zero-altered Poisson, and Hurdle distributions were tested for the variable Y. Based on the deviance information criterion (DIC), the Poisson distribution best fit the data.

The general statistical model is given below:

$$l=Xb+{Z}_{1}{u}_{1}+{Z}_{2}{u}_{2}+{Z}_{3}{u}_{3}+{Z}_{4}{u}_{4}+e$$

where $l$ is the vector of the latent variable for the studied trait (y); $y$ is the vector of the number of fungi (or bacteria); ${y}_{i}\sim Poisson({\lambda }_{i}=\mathrm{exp}\left({l}_{i}\right))$, where $i=\mathrm{1,2},\dots ,n$ and $n$ is the number of observations; $\lambda$ is the canonical parameter of this distribution (Poisson mean); $\mathrm{exp}$ is the inverse link function of the Poisson distribution; $b$ is the vector of systematic effects (mean, fermentation period, and treatment: CTRL, Inoc1, and Inoc2); ${u}_{1}$ is the vector of fungi type (or bacteria) species effect; ${u}_{2}$ is the vector for the interaction of fungi type (or bacteria) and fermentation period effect; ${u}_{3}$ is the vector for the interaction of fungi type (or bacteria) and inoculant effect; ${u}_{4}$ is the vector for the interaction of fungi type (or bacteria), fermentation period, and inoculant effect. Thus, it was defined that the vector of the latent variable assumes a multivariate normal distribution given by $l|b,{u}_{1},{u}_{2},{u}_{3},{u}_{4},{\sigma }_{1}^{2},{\sigma }_{2}^{2},{\sigma }_{3}^{2},{\sigma }_{4}^{2},{\sigma }_{e}^{2}\sim N(Xb+{Z}_{1}{u}_{1}+{Z}_{2}{u}_{2}+{Z}_{3}{u}_{3}+{Z}_{4}{u}_{4}, I{\sigma }_{e}^{2})$, where ${\sigma }_{1}^{2},{\sigma }_{2}^{2},{\sigma }_{3}^{2},{\sigma }_{4}^{2}$ and ${\sigma }_{e}^{2}$ are the variance components associated with ${u}_{1}, {u}_{2}$, ${u}_{3}, {u}_{4}$, and $e$ (error variance), respectively.

The assumed prior distributions for the model parameters were:

$$b\sim N\left({0,I10}^{8}\right)$$

$${u}_{1}|{\sigma }_{1}^{2}\sim N(0,I{\sigma }_{1}^{2})$$

$${u}_{2}|{\sigma }_{2}^{2}\sim N(0,I{\sigma }_{2}^{2})$$

$${u}_{3}|{\sigma }_{3}^{2}\sim N(0,I{\sigma }_{3}^{2})$$

$${u}_{4}|{\sigma }_{4}^{2}\sim N(0,I{\sigma }_{4}^{2})$$

The assumed prior distributions for the variance components were:

$${\sigma }_{1}^{2}\sim IG\left(\frac{{\alpha }_{1}}{2},\frac{{\alpha }_{1}{\beta }_{1}}{2}\right)$$

$${\sigma }_{2}^{2}\sim IG\left(\frac{{\alpha }_{2}}{2},\frac{{\alpha }_{2}{\beta }_{2}}{2}\right)$$

$${\sigma }_{3}^{2}\sim IG\left(\frac{{\alpha }_{3}}{2},\frac{{\alpha }_{3}{\beta }_{3}}{2}\right)$$

$${\sigma }_{4}^{2}\sim IG\left(\frac{{\alpha }_{4}}{2},\frac{{\alpha }_{4}{\beta }_{4}}{2}\right)$$

$${\sigma }_{e}^{2}\sim IG\left(\frac{{\alpha }_{e}}{2},\frac{{\alpha }_{e}{\beta }_{e}}{2}\right)$$

where IG is the inverse-gamma distribution, ${\alpha }_{1}$, ${\beta }_{1}$, ${\alpha }_{2}$, ${\beta }_{2}$, ${\alpha }_{3}$, ${\beta }_{3}$, ${\alpha }_{4}$, ${\beta }_{4},{\alpha }_{e}$, equal, to $0.001$ and ${\beta }_{e}$ are known constants called hyperparameters equal to ${10}^{8}.$

The statistical inference of the parameters from ($b,{u}_{1},{u}_{2},{u}_{3},{u}_{4},{\sigma }_{1}^{2},{\sigma }_{2}^{2},{\sigma }_{3}^{2},{\sigma }_{4}^{2},{\sigma }_{e}^{2}$) was based on posterior marginal distributions. In summary, random samples of the posterior marginal distributions were indirectly generated from the full conditional posterior distributions (f.c.p.d.) (likelihood function × the prior distribution of each parameter) through Markov chain Monte Carlo (MCMC) algorithms. If f.c.p.d. is characterized as a known family of probability distributions, the Gibbs sampler can be used. Thus, after a sufficiently large number of iterations, the values generated from f.c.p.d. were samples of posterior marginal distributions.

In the analysis, we used 2,000,000 iterations for the MCMC algorithms for the prior elicitation of different procedures, and the first 100,000 iterations were discarded as burn-in. Following the performance of every set of ten iterations (thin), a sample was retained to calculate the posterior statistics. Markov chain convergence was verified through Geweke’s diagnostic^29^.

By conducting these analyses, it was possible to verify which effects were important for the incidence of fungi and bacteria in corn and sorghum grains. The model with the smallest DIC was chosen; thus, those effects were considered relevant to explain the variable of interest (Supplementary Table S1). If the interaction between the type of inoculant and fermentation period was detected, we studied each factor within the other. Thus, the difference between the levels was detected through the credibility interval.

## Results

The chemical composition, pH, and microbial populations of the ground and rehydrated corn and sorghum grains are shown in Table 1. The average fermentation profile and microbial population of the rehydrated corn and sorghum grain silages are presented in Supplementary Table S2.

**Table 1:** Average chemical composition, pH and microbial populations (log cfu/g of fresh weight) of rehydrated corn and sorghum grains before fermentation.

Item	Corn grain			Sorghum grain			SEM
	CTRL	Inoc1	Inoc2	CTRL	Inoc1	Inoc2	
Dry matter (g kg^**−**1^ as fed)	674.9	679.8	688.6	690.1	686.5	684.0	1.83
Crude protein (g kg^−1^ DM)	76.8	77.4	74.8	101.8	101.0	100.1	2.60
Soluble protein (g kg^−1^ total N)	303.8	288.3	267.1	177.9	161.6	136.8	15.84
WSC^a^ (g kg^−1^ DM)	19.7	21.2	20.5	11.2	10.9	11.0	1.03
pH	5.88	5.77	5.91	6.35	6.39	6.40	0.055
Lactic acid bacteria	5.46	5.43	5.11	5.04	5.32	4.84	0.085
Enterobacteria	4.76	4.92	4.94	5.51	5.54	5.29	0.081
Fungi	5.75	6.42	5.76	4.29	4.65	4.59	0.186
IVDMD^b^ (%)	77.77	75.81	78.67	–	–	–	1.101

*CTRL* non-inoculated, *Inoc1* *Lactobacillus plantarum* and *Propionibacterium acidipropionici*, *Inoc2* *Lactobacillus buchneri.*

^a^Water soluble carbohydrate.

^b^In vitro dry matter digestibility.

^c^Standard error of the mean.

### Bacteriome of the silages

A total of 1,581,953 high-quality reads were generated, with 783,146 of these reads originating from the CG samples and 798,807 reads originating from SG on different days of fermentation. The number of sequences was standardized relative to the minimum number of 12,297 sequences obtained from a single sample. The bacterial communities in CG and SG samples are presented in Supplementary Tables S3 and S4, respectively. A total of 257 and 119 OTUs were detected in CG and SG samples, respectively. Rarefaction curves of OTUs at 97% sequence identity are shown in Supplementary Figure S1. The sequencing depth was sufficient to fully describe the diversity of the bacterial populations in silages as rarefaction curves reached a plateau for sequences.

### Diversity analysis

**Figure 1:**
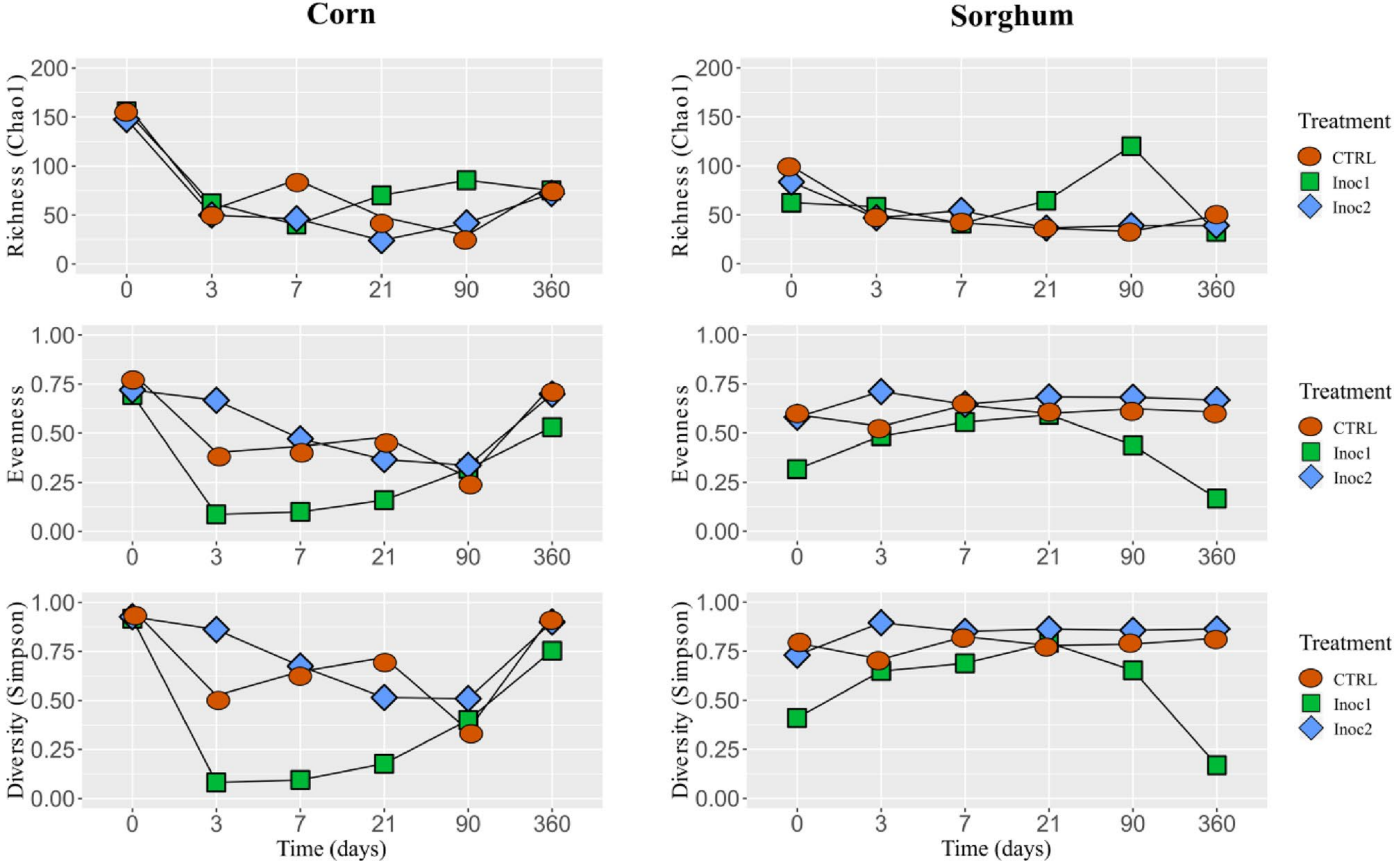
Chao 1 Richness, evenness and Simpson diversity of rehydrated corn and sorghum grain silages throughout the fermentation period (0, 3, 7, 21, 90 and 360 days). *CTRL* non-inoculated, *Inoc1* *Lactobacillus plantarum* and *Propionibacterium acidipropionici*, *Inoc2* *Lactobacillus buchneri*.

The Simpson diversity index of the rehydrated corn and sorghum grain silages is shown in Fig. 1. The initial diversity of the CG was similar between the treatments. There was a reduction in the diversity during the fermentation period, mainly for CG-Inoc1 silages, due to the lower evenness in these silages, as the Chao 1 richness decreased on the 3 days and remained similar between the treatments until the end of the fermentation period. The diversity of CG at 360 days increased, approaching the initial values.

Numerically, SG samples had a lower initial diversity index than CG samples, and the diversity index increased from day 3 onwards. The diversity responses of the silages in the different treatments were similar, except in SG-Inoc1, which had lower initial numbers and reduced values after 90 days of fermentation due to the reduction in evenness.

Comparing the effect of inoculation on bacterial abundance within each fermentation period (Table 2), according to the Poisson model estimates, the highest bacterial abundance was found in CG-CTRL silages on 0, 7, and 90 days of fermentation; however, the lowest value was observed for these silages after 21 days of fermentation. No difference between the inoculants was found after 360 days of fermentation. For SG-silages, inoculation promoted differences in bacterial abundance only on day 0 of fermentation, resulting in SG-Inoc2 silages with higher abundance and SG-CTRL with intermediate values, followed by SG-Inoc1 silages.

**Table 2:** Study of inoculants within each fermentation period for the bacterial abundance of ground and rehydrated corn and sorghum grain silages.

Grain	Inoculant	Fermentation period (days)											
		0		3		7		21		90		360	
		Mean	CV	Mean	CV	Mean	CV	Mean	CV	Mean	CV	Mean	CV
Corn	CTRL	7.22^a^	0.66	5.18^b^	0.92	5.63^a^	0.45	5.18^b^	0.71	7.20^a^	1.00	5.13	0.91
	Inoc1	5.12^b^	0.67	5.14^b^	0.97	5.10^b^	0.53	5.35^a^	0.70	5.21^b^	0.96	5.14	0.90
	Inoc2	5.12^b^	0.67	5.53^a^	0.92	5.11^b^	0.51	5.30^a^	0.70	5.19^b^	0.98	5.17	0.85
Sorghum	CTRL	24.00^b^	0.28	22.02	1.10	22.02	1.21	21.99	1.26	22.01	1.76	25.45	60.08
	Inoc1	22.52^c^	0.29	22.01	1.10	22.01	1.22	22.01	1.23	22.01	1.80	24.02	66.00
	Inoc2	25.86^a^	0.27	22.02	1.08	22.01	1.23	22.00	1.24	22.01	1.74	87.82	63.02

*CTRL* non-inoculated, *Inoc1* *Lactobacillus plantarum* and *Propionibacterium acidipropionici*, *Inoc2* *Lactobacillus buchneri.*

^a−b^Means followed by different letters in the column differ for each combination between inoculant and grain within each fermentation period by credibility interval.

Analysis of the behavior of bacterial abundance over the fermentation period for each inoculation (Table 3) revealed differences in bacterial abundance throughout the fermentation period for all inoculants in both grain silages. The abundance of SG silages was higher in unfermented materials (day 0) than in others, regardless of the inoculation, whereas in CG silages, a pattern was not observed.

**Table 3:** Study of each inoculant throughout the fermentation period for the bacterial abundance of ground and rehydrated corn and sorghum grain silages after 0, 3, 7, 21, 90 and 360 days of fermentation.

Grain	Inoculant	Fermentation period (days)					
		0	3	7	21	90	360
Corn	**CTRL**						
	Mean	7.66^a^	5.22^c^	6.18^b^	5.17^c^	6.81^a,b^	5.13^c^
	CV	0.40	0.44	0.43	0.46	0.42	0.46
	**Inoc 1**						
	Mean	5.16^b^	5.13^b^	5.13^b^	5.31^a^	5.17^b^	5.15^b^
	CV	0.78	0.83	0.83	0.76	0.77	0.80
	**Inoc 2**						
	Mean	5.19^b,d^	5.74^a^	5.15^c,d^	5.33^b^	5.19^b,d^	5.33^b^
	CV	0.66	0.63	0.69	0.64	0.66	0.64
Sorghum	**CTRL**						
	Mean	26.40^a^	22.11^b^	22.10^b^	21.90^b^	21.95^b^	22.22^b^
	CV	1.01	0.97	0.96	1.01	0.98	0.98
	**Inoc1**						
	Mean	26.11^a^	22.54^b^	22.53^b^	22.84^a^	22.25^b^	22.55^b^
	CV	1.14	1.07	1.06	1.06	1.05	1.08
	**Inoc2**						
	Mean	35.98^a^	22.14^b^	22.13^b^	22.10^b^	21.91^b^	22.15^b^
	CV	0.88	0.83	0.83	0.82	0.83	0.82

*CTRL* non-inoculated, *Inoc1* *Lactobacillus plantarum* and *Propionibacterium acidipropionici*, *Inoc2* *Lactobacillus buchneri.*

^a−e^Means followed by different letters in the line differ by credibility interval.

### Taxonomic composition—phylum and class

Analyses of the relative abundance of bacterial communities revealed the presence of nine and six phyla in CG and SG, respectively (Supplementary Figure S2). The phyla *Actinobacteria*, *Bacteroides*, *Deinococcus-Thermus*, *Firmicutes*, and *Proteobacteria* were found in both grains. The phylum *Cyanobacteria* was found in SG, and *Acidobacteria*, *Chloroflexi*, *Planctomycetes*, and *Verrucomicrobia* were found only in CG samples.

The predominance (> 80%) of *Proteobacteria* was observed in both grains at the beginning of the fermentation (day 0), except in CG-CTRL and CG-Inoc1, which had 51% of *Proteobacteria* and 61% of *Actinobacteria,* respectively. In all CG silages, *Firmicutes* phylum dominated (> 84%) the fermentation from 3 to 90 days after ensiling. There was a tendency in the bacterial community to return to its initial diversity at 360 days of fermentation, with the replacement of *Firmicutes* by *Proteobacteria* and *Actinobacteria*.

**Figure 2:**
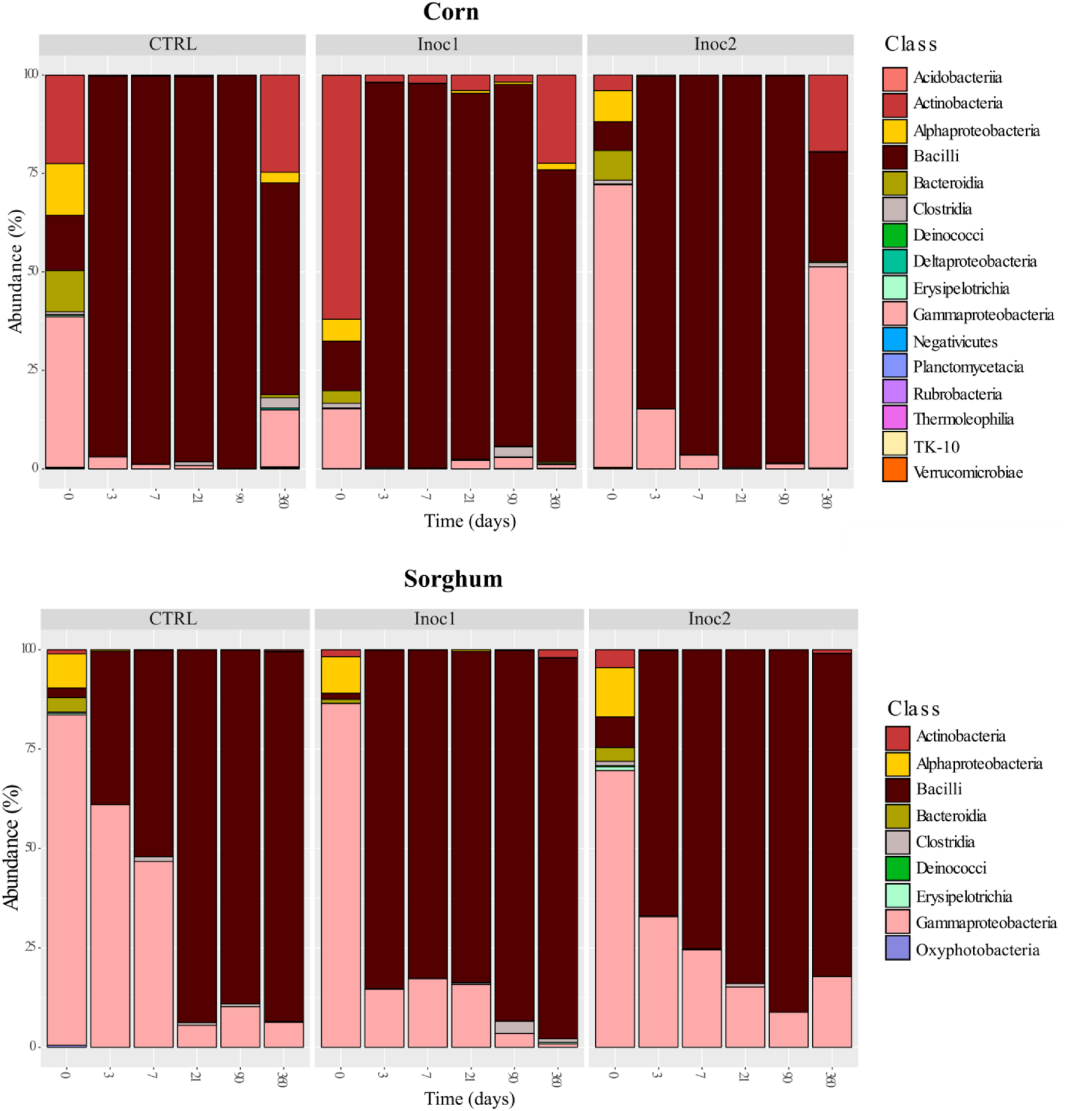
Classes taxonomic profiles of bacterial communities of rehydrated corn and sorghum grain silages after 0, 3, 7, 21, 90 and 360 days of fermentation. *CTRL* non-inoculated, *Inoc1* *Lactobacillus plantarum* and *Propionibacterium acidipropionici*, *Inoc2* *Lactobacillus buchneri*.

As observed for phyla, the number of bacterial classes found in the CG samples was higher than that in the SG samples (16 vs. 9) (Fig. 2). The classes *Actinobacteria*, *Alphaproteobacteria*, *Bacilli*, *Bacteroidia*, *Clostridia*, *Deinococci*, *Erysipelotrichia*, and *Gammaproteobacteria* were commonly found in both grains. *Oxyphotobacteria* was present only in SG, while *Acidobacteria*, *Deltaproteobacteria*, *Negativicutes*, *Planctomycetacia*, *Rubrobacteria*, *Thermoleophilia*, TK10, and *Verrucomicrobiae* were found only in CG samples. The *Bacilli* and *Gammaproteobacteria* classes were the main representatives of the phyla *Firmicutes* and *Proteobacteria*, respectively. During the fermentation period, the changes observed in these two main phyla were mainly the result of increased or reduced numbers of these two classes.

Both microbial inoculants favored *Bacilli* growth in CG and SG silages; however, bacteria present in Inoc1 were more effective in promoting changes in the bacterial population of both grain silages as early as the 3rd day of fermentation.

### Taxonomic composition—genus

**Figure 3:**
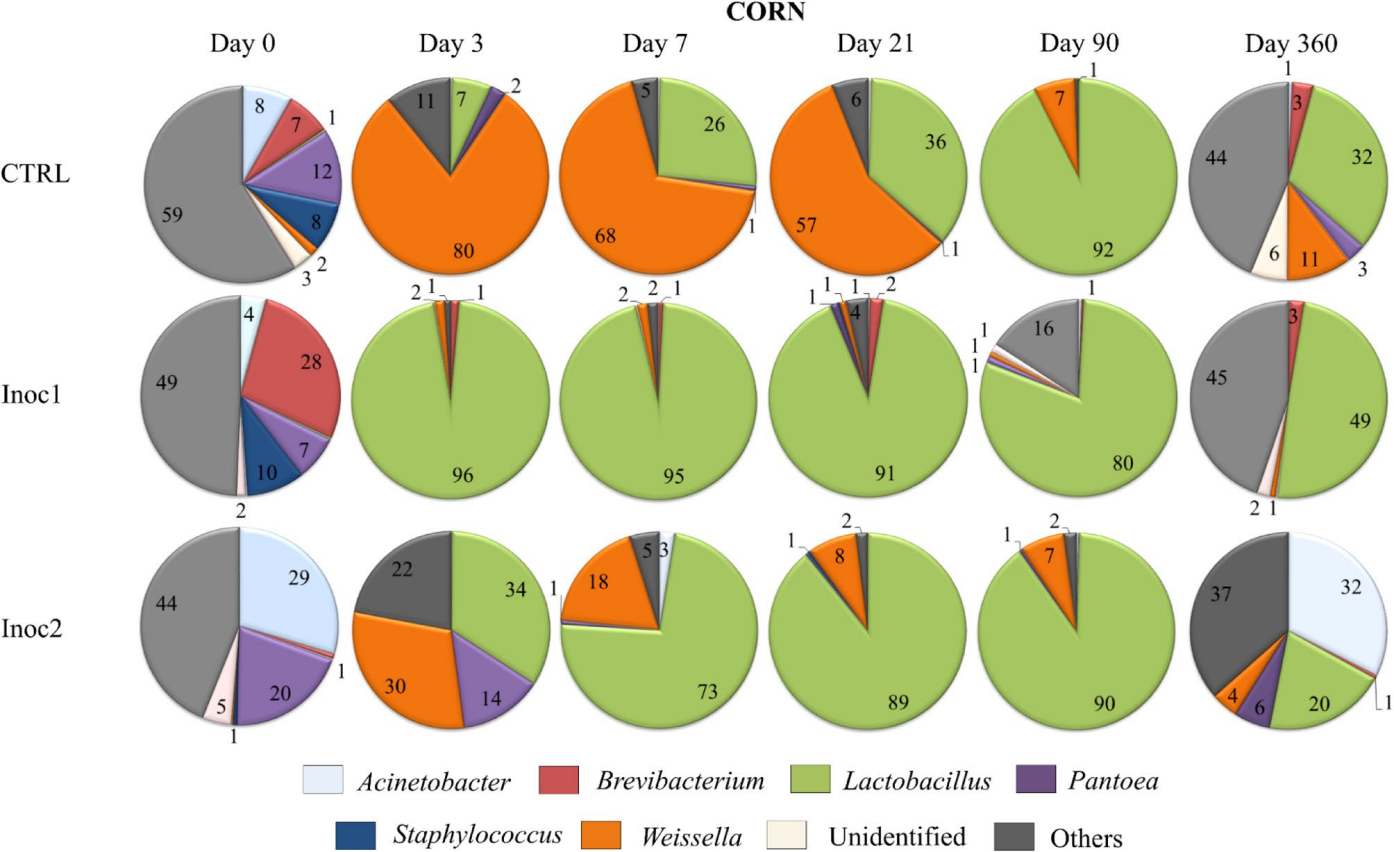
Main genera dynamics (%) of bacterial communities of rehydrated corn grain silages after 0, 3, 7, 21, 90 and 360 days of fermentation. *CTRL* non-inoculated, *Inoc1* *Lactobacillus plantarum* and *Propionibacterium acidipropionici*, *Inoc2* *Lactobacillus buchneri*.

The dynamics of the main genera in CG silages are presented in Fig. 3. Although the *Bacilli* class was found predominantly in both grains during the intermediate periods of fermentation, the succession of the genera representing this class was distinguished among the silages. The initial genus composition of the CG was uneven among the treatments. In the CS-CTRL, the fermentation from the 3rd day was predominantly by *Weissela*, with gradual replacement by the *Lactobacillus* genus, which represented 93% of the genera at 90 days. Bacteria in Inoc1 induced the predominance (> 80%) of *Lactobacillus* from 3 to 90 days of fermentation.

Although *Lactobacillu*s represented ≅ 90% of the bacteria in CS-Inoc2 at 21 and 90 days, the inoculant was not as efficient as Inoc1 in preventing the development of *Weissella* and other genera on 3 days and 7 days of fermentation. Increased evenness was observed in the silages of all treatments at 360 days, resulting in the substitution of *Lactobacillus*. The CS-Inoc1 silages had the highest *Lactobacillus* counts (50%), evidencing the greater efficiency of Inoc1 in maintaining the equilibrium of the bacterial population in the final period evaluated. CS-Inoc2 silages were the ones that mostly tended to return to the initial population profile, with a relative abundance of 32% for *Acinetobacter* and 37% for other genera.

**Figure 4:**
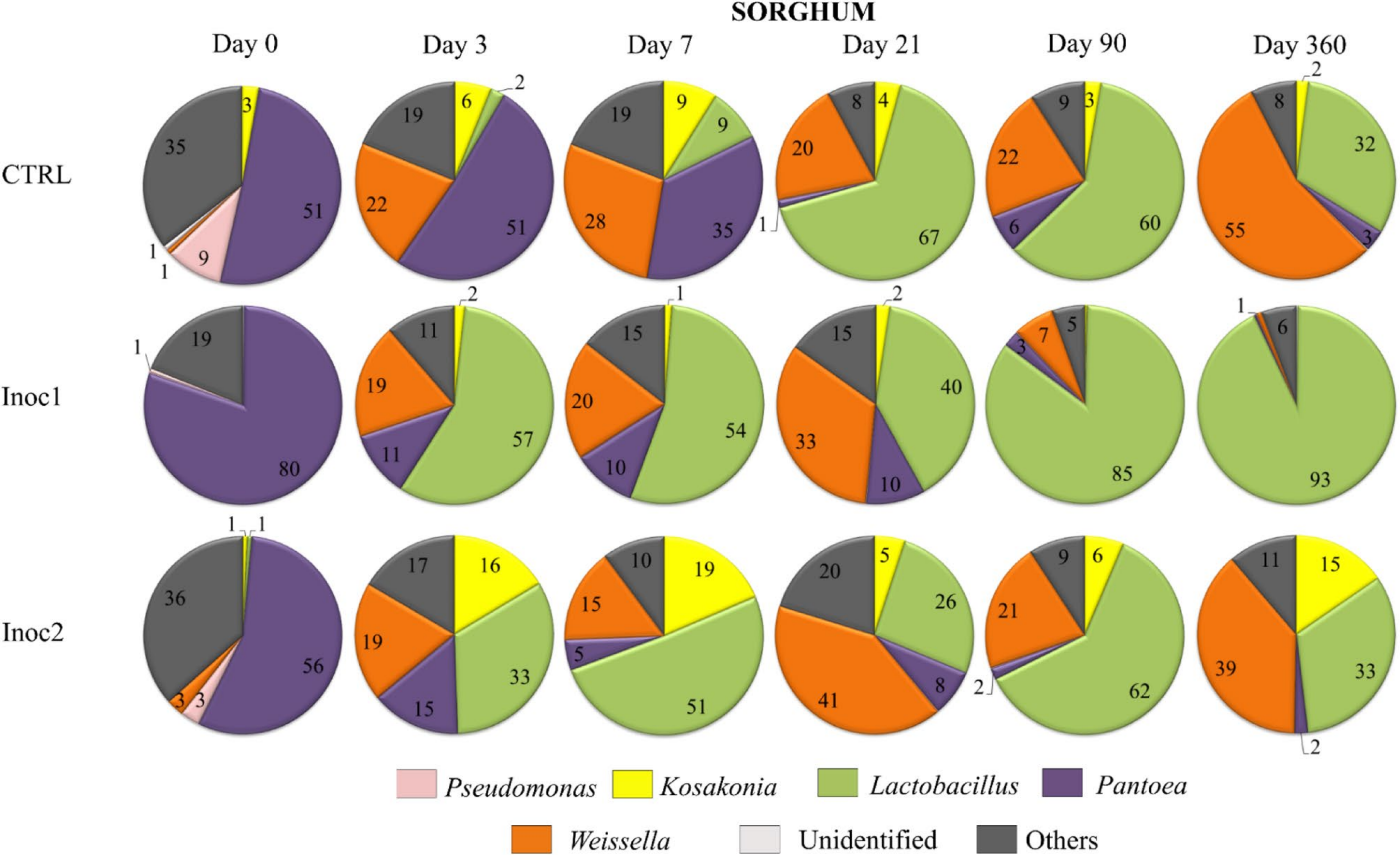
Main genera dynamics (%) of bacterial communities of rehydrated sorghum grain silages after 0, 3, 7, 21, 90 and 360 days of fermentation. *CTRL* non-inoculated, *Inoc1*: *Lactobacillus plantarum* and *Propionibacterium acidipropionici*, *Inoc2* *Lactobacillus buchneri.*

The dynamics of the genera profile in SG silages are shown in Fig. 4. The bacterial community changes in the inoculated SG silages were more complex than those in the CG silages, indicating the more complicated interactions between the bacterial flora. Unlike CG, whose initial populations were heterogeneous, SG had lower initial richness and evenness, which reflected the predominance (51–80%) of the *Pantoea* genus, mainly in SG-Inoc1 silages (80%). Throughout the fermentation period, gradual but not total genus substitutions occurred by different proportions of *Weissella, Lactobacillus*, and bacteria belonging to the *Enterobacteriaceae* family.

Bacterial replacement was less pronounced in SG silages than in CG silages during the fermentation period. A high percentage (> 85%) of *Lactobacillus,* as observed in CG-Inoc1 during the initial stages of fermentation, occurred only in SG-Inoc1 silages from 90 days onwards. SG-Inoc2 had similar bacterial succession as SG-CTRL from 90 days onwards, with the presence of different genera in the bacterial community. As observed in CG at 360 days, there were changes in the bacterial taxonomic composition, mainly the replacement of *Lactobacillus* by *Weissella* in SG-CTRL silages and *Weissella* and *Kosakonia* in SG-Inoc2. However, the changes in SG were mainly at the family level, whereas in the CG silages, substitutions were also observed at the phylum level. The SG-Inoc1 sample presented the greatest stability of the bacterial composition at 360 days, with 93% represented by *Lactobacillus.*

### Mycobiome of the silages

In total, 2,569,416 high-quality reads were obtained, of which 1,587,182 reads and 982,334 reads originated from CG and SG samples, respectively, on different days of fermentation. The quality of the sequences present in SG-Inoc2 at 360 days was insufficient to identify the fungal community. The number of sequences was standardized relative to the minimum number of 2863 sequences obtained from a single sample.

The fungal communities in CG and SG are presented in Supplementary Tables S5 and S6, respectively. A total of 71 and 109 OTUs were detected in CG and SG samples, respectively. Rarefaction curves of OTUs at 97% sequence identity are shown in Supplementary Figure S3. The sequencing depth was sufficient to fully describe the diversity of the fungal populations in silages, as rarefaction curves reached a clear plateau for sequences.

### Diversity analysis

**Figure 5:**
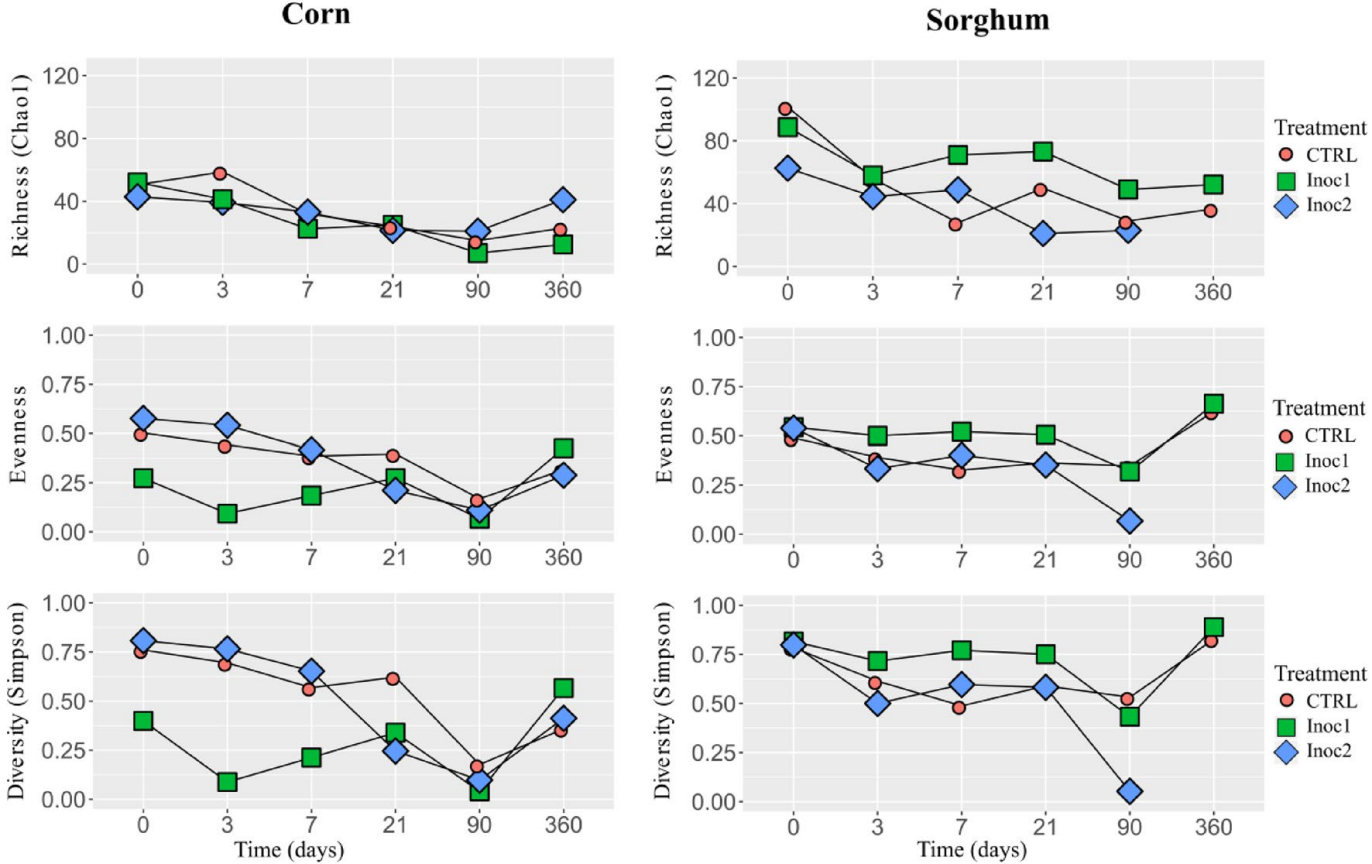
Fungal Chao 1 Richness, evenness, Simpson diversity of rehydrated corn and sorghum grain silages throughout the fermentation period (0, 3, 7, 21, 90 and 360 days). *CTRL* non-inoculated, *Inoc1* *Lactobacillus plantarum* and *Propionibacterium acidipropionici*, *Inoc2* *Lactobacillus buchneri*.

The Simpson diversity index of corn and sorghum grain silages is shown in Fig. 5. There was fluctuation in mycobiome diversity throughout the fermentation period in all treatments. Numerically, CG-Inoc1 silage had lower diversity at the beginning of the fermentation than other CG silages. At 90 days, there was a drop in the diversity of all treatments, which then increased again after 360 days, mainly due to variation in evenness. In general, the diversity of SG samples was higher than that of the CG samples. As observed in CG at 90 days, there was a reduction in diversity in all SG treatments, especially in SG-Inoc2 silage, which increased again after 360 days of fermentation.

According to the Poisson model estimates, when analyzing the effect of inoculants on fungal abundance within each fermentation period (Table 4), the highest values were observed for CG-CTRL, and the lowest values were observed for CG-Inoc2 silages after 0 days and 3 days of fermentation. However, no difference was observed in the other fermentation periods, and no difference in the fungal abundance of SG-silages was observed at 0 days and 360 days of fermentation. However, SG-Inoc1 silages showed the highest values after 3 and 21 days of fermentation. The lowest abundances were observed in the SG-CTRL and SG-Inoc2 silages on days 7 and 90, respectively.

**Table 4:** Study of inoculants within each fermentation period for the fungal abundance of ground and rehydrated corn and sorghum grain silages after 0, 3, 7, 21, 90 and 360 days of fermentation.

Grain	Inoculant	Fermentation period (days)											
		0		3		7		21		90		360	
		Mean	CV	Mean	CV	Mean	CV	Mean	CV	Mean	CV	Mean	CV
Corn	CTRL	799.42^a^	305.07	118.35^a^	264.26	80.27	69.39	66.72	67.00	88.02	152.22	65.45	18.83
	Inoc1	116.44^a,b^	365.23	66.01^a,b^	204.79	66.27	78.00	65.04	34.01	66.43	102.38	65.21	13.56
	Inoc2	66.93^b^	159.06	65.17^b^	243.44	66.34	32.40	67.94	89.04	65.78	141.33	67.32	53.29
Sorghum	CTRL	11.58	0.39	23.09^b^	0.50	24.51^b^	0.56	25.00^b^	0.47	26.12^a^	0.88	28.18	56.70
	Inoc1	11.59	0.39	23.21^a^	0.49	24.82^a^	0.51	25.40^a^	0.45	26.30^a^	0.88	35.17	106.32
	Inoc2	11.56	0.39	23.08^b^	0.51	24.64^a^	0.53	24.85^c^	0.51	26.07^b^	0.94	33.08	42.97

*CTRL* non-inoculated, *Inoc1* *Lactobacillus plantarum* and *Propionibacterium acidipropionici*, *Inoc2* *Lactobacillus buchneri.*

^a−b^Means followed by different letters in the column differ for each combination between inoculant and grain within each fermentation period by credibility interval.

The effects of each inoculation on fungal abundance throughout the fermentation period in both grains are shown in Table 5. Fungal abundance did not differ between CG-Inoc1, CG-Inoc2, SG-CTRL, and SG-Inoc1 silages and decreased in CG-CTRL and SG-Inoc2 silages throughout the fermentation period.

**Table 5:** Study of each inoculant throughout the fermentation period for the fungal abundance of ground and rehydrated corn and sorghum grain silages after 0, 3, 7, 21, 90 and 360 days.

Grain	Inoculant	Fermentation period (days)					
		0	3	7	21	90	360
Corn	**CTRL**						
	Mean	65.77^a^	65.28^a,d^	65.62^a,b^	65.07^b,d^	64.99^d^	65.02^c,d^
	CV	0.91	0.93	0.91	0.96	1.00	0.99
	**Inoc1**						
	Mean	65.07	65.07	65.07	65.06	65.06	65.07
	CV	1.72	1.74	1.72	1.78	1.76	1.73
	**Inoc2**						
	Mean	65.25	65.02	66.43	68.86	65.15	75.84
	CV	12.95	7.41	18.07	9.55	14.86	50.21
Sorghum	**CTRL**						
	Mean	23.28	22.71	22.48	22.76	22.52	22.49
	CV	0.95	0.96	0.97	0.95	0.98	0.94
	**Inoc1**						
	Mean	23.21	23.20	23.29	24.03	23.07	23.53
	CV	0.84	0.83	0.84	0.83	0.83	0.83
	**Inoc2**						
	Mean	23.13^a^	22.80^a,c^	22.89^a^	22.69^b,c,e^	22.67^d,e^	–
	CV	0.81	0.83	0.82	0.87	0.91	–

*CTRL* non-inoculated, *Inoc1* *Lactobacillus plantarum* and *Propionibacterium acidipropionici*, *Inoc2* *Lactobacillus buchneri.*

^a−e^Means followed by different letters in the line differ by credibility interval.

### Taxonomic composition—phylum and class

The phyla *Ascomycota*, *Basidiomycota*, and *Mucoromycota* were found in both grains. Ascomycota was predominant in all samples, with few variations in the presence of the other phyla (Supplementary Figure S4).

**Figure 6:**
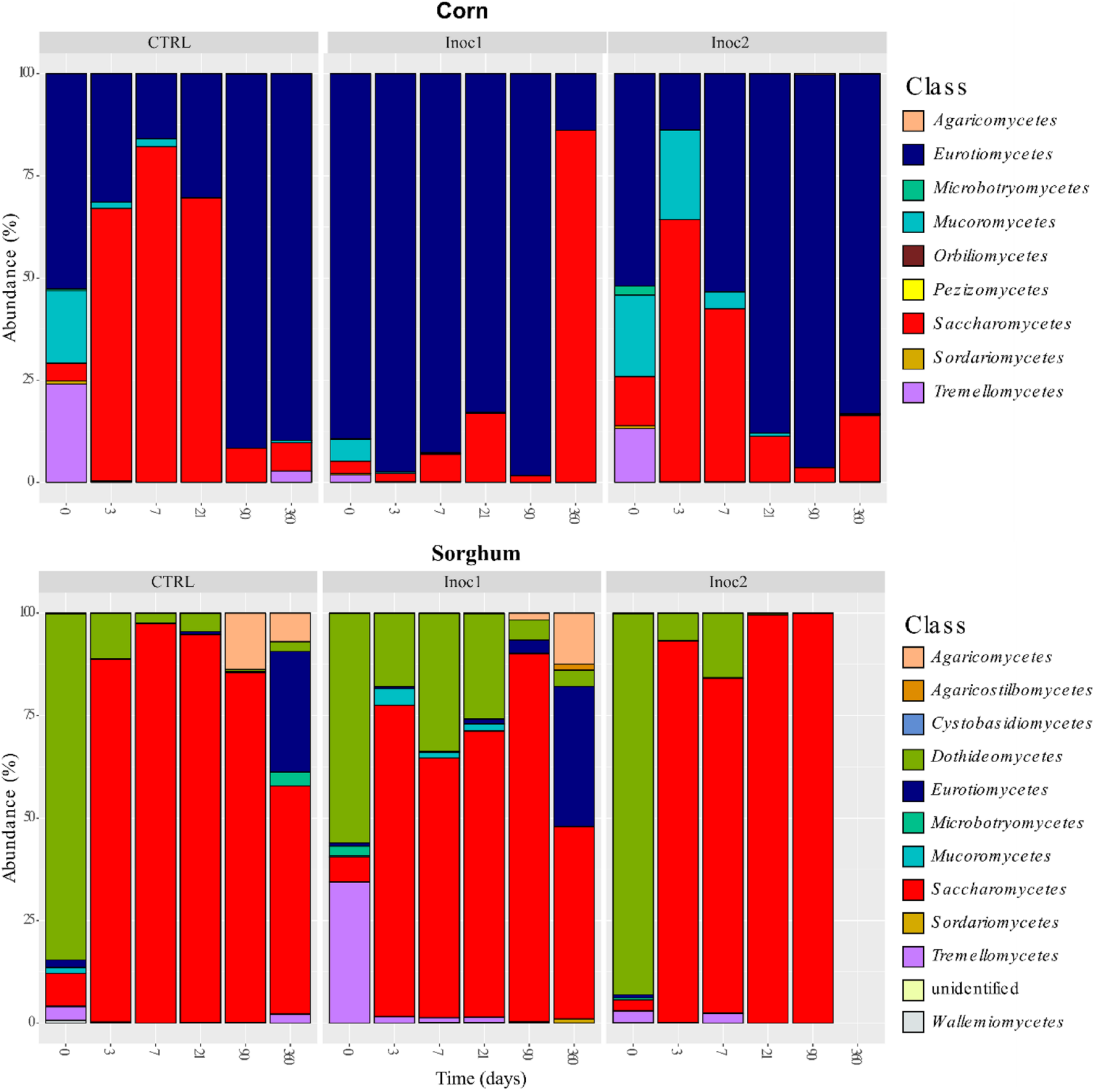
Class taxonomic profiles of fungal communities of rehydrated corn and sorghum grain silages after 0, 3, 7, 21, 90 and 360 days of fermentation. *CTR *non-inoculated, *Inoc1* *Lactobacillus plantarum* and *Propionibacterium acidipropionici*, *Inoc2* *Lactobacillus buchneri*.

Unidentified fungi and the other eleven classes were found in both grains (Fig. 6). The classes *Agaricomycetes, Dothideomycetes, Eurotiomycetes, Microbotryomycetes, Mucoromycetes, Saccharomycetes, Sordariomycetes, Tremellomycetes,* and *Wallemiomycetes* were found in both grains, *whereas Orbiliomycetes* and *Pezizomycetes* were found only in CG samples, and *Agaricostilbomycetes* and *Cystobasidiomycetes* were found in SG samples. *Saccharomycetes* and *Eurotiomycetes* were predominantly found in CG, whereas *Dothideomycetes* and *Saccharomycetes* were the main classes found in SG fungal fermentation. In CG-CTRL and CG-Inoc2 silages, approximately half of the initial populations were microorganisms belonging to the *Eurotiomycetes* class, with smaller proportions of *Sordariomycetes* and *Tremellomycetes*. On day 3 of fermentation, there was a large increase in *Saccharomycetes* in both silages, with gradual replacement by *Eurotiomycetes,* whose predominance (> 80%) was extended up to 360 days. *Dothideomycetes* accounted for 56–92% of the initial population in SG samples. *Tremellomycetes* were also present in significant amounts prior to fermentation in the SG-Inoc1 silage. The initial populations were replaced by *Saccharomycetes*, with dominance extending up to 90 days in all silages. At 360 days, influential amounts of *Eurotiomycetes* replaced the *Saccharomycetes* microorganism*s* in the SG-CTRL and SG-Inoc1 silages.

### Taxonomic composition—genus

**Figure 7:**
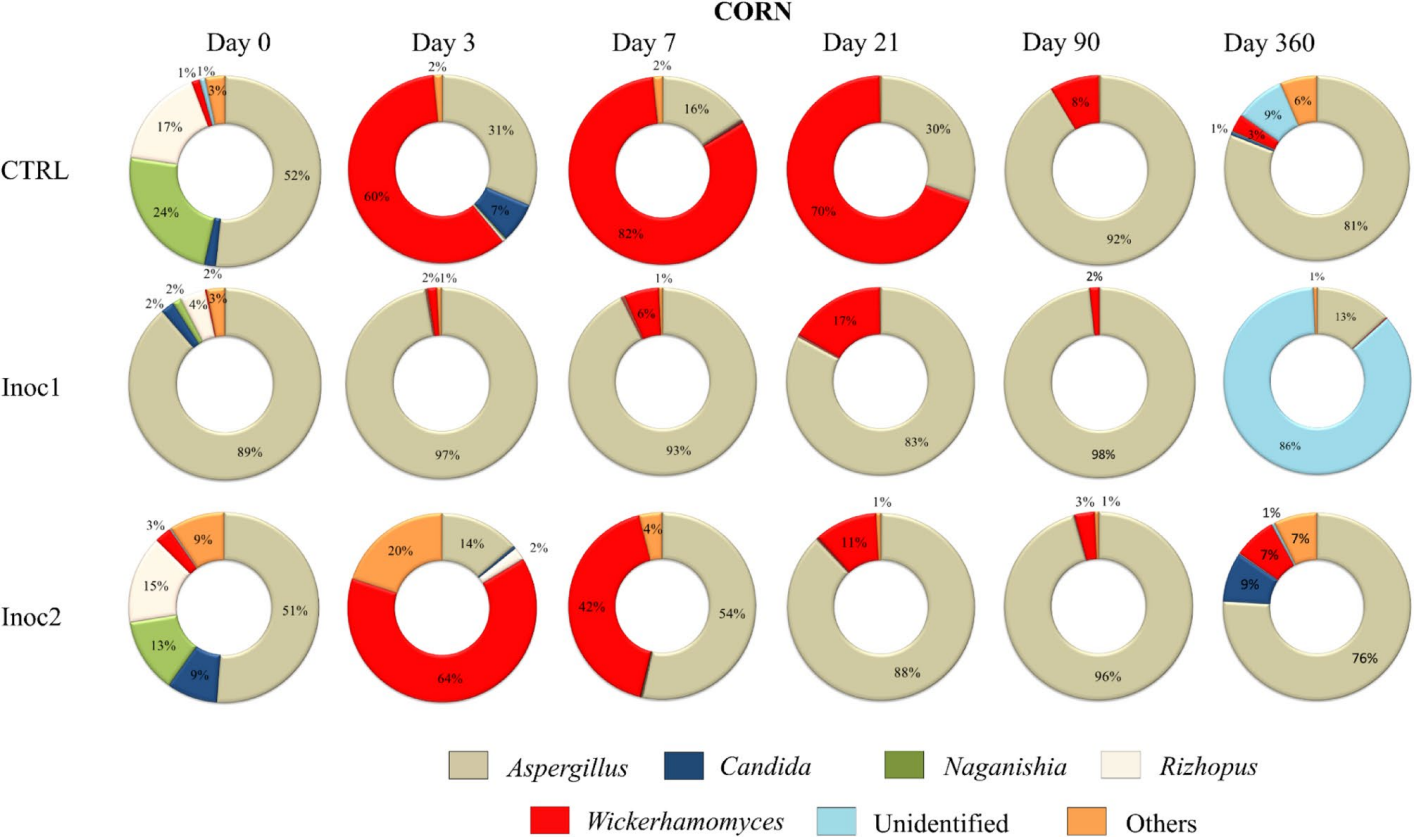
Main genera dynamics (%) of fungal communities of rehydrated corn grain silages after 0, 3, 7, 21, 90 and 360 days of fermentation. *CTRL* non-inoculated, *Inoc1* *Lactobacillus plantarum* and *Propionibacterium acidipropionici*, *Inoc2* *Lactobacillus buchneri*.

The main genera dynamics of corn grain silages are shown in Fig. 7. *Aspergillus* spp. represented 51–89% of the initial population in CG samples. Its predominance (89%) in CG-Inoc1 silage at 0 days resulted in the lowest initial diversity in these silages and was also responsible for the sharp reduction in diversity in all CG silages at 90 days. In CG-CTRL silages, as early as 3 days after fermentation, there was a growth of *Wickerhamomyces* (60%) yeasts that dominated the fermentation until 21 days. *Aspergillus* returned to dominate from 90 days until the end of the fermentation. A similar response was observed in CG-Inoc2 silage; however, *Aspergillus* already represented 54% and 88% of the genera at 7 and 21 days, respectively. Bacteria present in Inoc1 resulted in fermentation with a predominance of *Aspergillus* up to 90 days. However, at 360 days, 86% of the genera were represented exclusively by unidentified yeasts belonging to *Saccharomycetales.*

**Figure 8:**
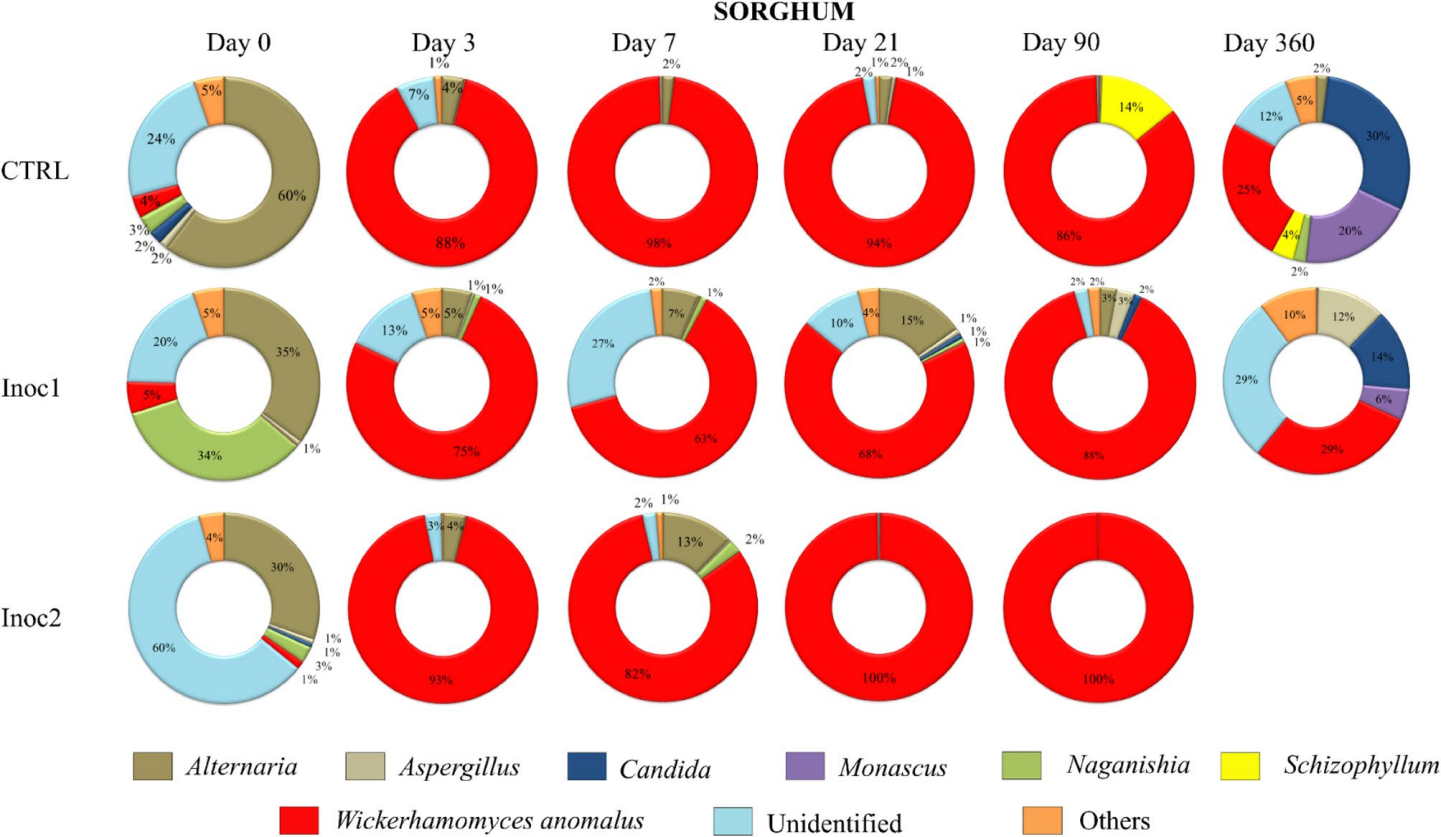
Main genera dynamics (%) of fungal communities of rehydrated sorghum grain silages after 0, 3, 7, 21, 90 and 360 days of fermentation. *CTRL* non-inoculated, *Inoc1* *Lactobacillus plantarum* and *Propionibacterium acidipropionici*, *Inoc2* *Lactobacillus buchneri*.

The main genera dynamics of sorghum grain silages are shown in Fig. 8. Greater participation of different genera was observed in the initial SG samples than in the CG samples. *Alternaria* spp. accounted for 30–60% of the initial population of the silages. Moreover, larger quantities of unidentified fungi belonging to *Pleosporales* order were observed, mainly in SG-Inoc2 samples (60%). *Wickerhamomyces anomalus* predominated the population of all silages at intermediate periods of fermentation, with a small participation of other genera. As previously observed, the diversity of SG-CTRL and SG-LM silages at 360 days increased due to increased evenness values, reflecting the greater participation of other genera, such as *Monascus, Candida,* and *Aspergillus,* during silage fermentation.

Some OTUs in SG samples were classified at the species level and included *Amylostereum chailletii, Aspergillus flavus, Exserohilum turcicum, Hyphoderma setigerum, Kwoniella heveanensis, Kwoniella mangrovensis, Monascus purpureus, Mucor circinelloides, Nigrospora oryzae, Phialemoniopsis curvata, Resinicium saccharicola, Rhizopus arrhizus, Rhodotorula diobovata, Rhodotorula mucilaginosa, Schizophyllum commune, Setophoma sacchari, Sterigmatomyces halophilus, Strelitziana eucalypti, Wallemia ichthyophaga, W. anomalus, Xeromyces bisporus,* and *Zygoascus hellenicus*.

## Discussion

The similar initial bacterial communities in SG reflected the minimal immediate impact of the treatments on the microbial community at the onset of the time course and the absence of significant differences in exogenous microbial contaminants that could alter the microbial make-up^30^. The predominance of *Proteobacteria* in the raw material was also reported by Romero et al.^31^ and McGarvey et al.^32^ in corn and alfalfa samples, respectively. Moreover, the presence of *Actinobacteria* in the initial composition of CG-Inoc1 was much higher (61%) than that observed by Romero et al*.*^31^ in corn samples (4.5%).

The high abundance of *Firmicutes* and low abundance of *Proteobacteria* in both grain silages after fermentation was also observed in wilted whole-crop oats ensiled for 217 days^33^ and alfalfa silage inoculated with different microbial inoculants^13^. Hence, the environmental conditions during ensiling contribute to the growth of the *Firmicutes* phylum^14^, ensuring the conservation of small grain silages^34^.

In grass silage, most reads assigned to the phylum *Firmicutes* were related to the order *Lactobacillales*, comprising the three most prevalent genera *Lactobacillus, Lactococcus*, and *Weissella*^35^. In our study, the *Acinetobacter* genus also had influential quantities in CG-Inoc2 silage at 360 days. *Acinetobacter* spp. are aerobic, non-fermenting bacteria found in different environments^36^. Some species can survive in an anaerobic environment in the presence of acetate as a substrate^37^ and require energy from carbohydrate degradation^38^. The increased abundance of *Acinetobacter* in CG-Inoc2 silage at 360 days may have resulted from the increased acetate concentration produced by the inoculated *L. buchneri* strain, as observed at 90 days of fermentation (Supplementary Table S2). This may partly explain the small, though important, dry matter (DM) losses sometimes observed in silages that had been treated with these heterofermentative bacteria during ensiling^39^.

The reduction in bacterial diversity in CG silages and SG silages inoculated with Inoc1 from 3 and 90 days of fermentation, respectively, can be explained by the dominance of *Lactobacillus* when inoculant containing *L. plantarum* was applied. Low bacterial diversity resulting from the high abundance of *Lactobacillus* was also reported by Ogunade et al.^40^ in corn silage. Hence, the more abundant the dominant bacterium, the less diverse the microbial community^41,42^. According to McDonald et al*.*^43^, after anaerobic fermentation, the complex microbial communities of the raw materials are gradually replaced by lactic acid bacteria (LAB), and *Lactobacillus* can become a dominant genus in successful silages. Indeed, as shown in Table 1 and Supplementary Table S2, LAB culture-dependent counts increased, and enterobacteria and fungi reduced gradually throughout the fermentation period in all treatments.

The increased *Lactobacillus* abundance in silages could be due to their ability to use starch or cellulose in addition to water-soluble carbohydrates (WSC), because the expression of amylases and both 1–4 and 1–6 glucosidases in amylolytic LAB isolated from sorghum has been previously reported by Velikova et al*.*^44^. Muck^45^ reported that the current strains of *L. buchneri* are rather slow compared to those of other species. Therefore, other LAB may perform the primary work of fermentation, and after active fermentation, the *L. buchneri* strains slowly convert lactic acid to acetic acid. This means that their effect on aerobic stability may take a while to be observed, typically 45–60 days after fermentation.

In our study, the *Lactobacillus* genus represented 73% and 51% of the microbial population in CG-Inoc2 and SG-Inoc2 silages, respectively, at 7 days of fermentation. Data from the fermentation profile of silages (Supplementary Table S2) suggest that the production of acetic acid by *L. buchneri* occurred after 90 days, mainly in SG-Inoc2 silages. Therefore, we can speculate that other species of the *Lactobacillus* genus functioned as starter cultures during the initial fermentation of these silages, as suggested by Muck^45^. Although *P. acidipropionici* was present in the composition of Inoc1, interestingly, no representatives of this genus were found in the silages of either grain. These propionic bacteria belong to the class *Actinobacteria*, which was mainly present in the initial population of CG-Inoc1 and was represented by different genera, including *Brevibacterium, Streptomyces,* and *Arthrobacter*. According to Filya et al*.*^46^, the combination of *P. acidipropionici* and *L. plantarum* does not appear to be promising in protecting wheat, sorghum, and corn silages from aerobic exposure. In general, *Propionibacterium* has been effective in situations where the decline in pH is low and/or when the final pH of silage is relatively high (> 4.2–4.5)^47^, which was observed in our study, mainly in SG silages. The absence of this genus in all samples was unexpected, particularly in the recently inoculated samples (day 0).

Bacteria belonging to the genus *Weissella* are strictly heterofermentative, producing a mixture of lactate and acetate as the major end-products of sugar metabolism^48,49^. Some species have been isolated from a wide range of sources such as soil, fresh vegetables, meat, fish, fermented silage, and foods^50–52^. In agreement with Muck^53^, who suggested that the biggest deviation in the microbial community appears to be in corn silage in warm climates, where *Weissella and Leuconostoc* species contributed to the early stages of fermentation, in our study, the fermentation of CG-CTRL silages until 21 days of fermentation was also predominated by *Weissella* spp. In addition, it was influenced by the presence of this genus in the SG silages throughout the fermentation period.

Pang et al*.*^54^ indicated that several *Weissella* spp. could improve silage quality. Furthermore, Ndagano et al*.*^55^ reported the production of acetate and other antifungal compounds, such as 3-hydroxy fatty acids and phenyllactate, by *Weissella paramesenteroides* isolated from fermented *Manihot esculenta*. However, Cai et al*.*^56^ concluded that heterofermentative strains of *W. paramesenteroides* did not improve silage quality and may cause some fermentation loss because of the greater growth of aerobic bacteria and clostridia, high pH, butyric acid, and ammonia nitrogen concentrations and lower lactic acid content in alfalfa and Italian ryegrass silages. The effect of *Weissella* genus on silage fermentation is still contradictory and unclear.

Spoiled silages are sometimes characterized by high levels of the *Enterobacteriaceae* family^57^. Several species, including *Salmonella* and *Escherichia coli*, are closely linked to human and animal diseases. The influential presence of *Enterobacteriaceae* in SG silages was mainly represented by the genus *Pantoea*, followed by smaller proportions of *Kosakonia*. The genus *Pantoea* is a highly diverse group whose members are found in aquatic and terrestrial environments and in association with plants, insects, humans, and animals^58^. McGarvey et al.^32^ also reported that *Pantoea* is one of the most commonly found genera in the bacterial community of alfalfa. The presence of this genus throughout the fermentation period in SG silages contradicts the findings of Si et al.^13^, in which the genus disappeared by 30 days in alfalfa silage. The authors also reported that these bacteria were negatively correlated with acetic acid and ammonia nitrogen concentrations and positively correlated with the WSC content. Jacxsens et al.^59^ reported that *Pantoea agglomerans* can ferment sugars to acids under anaerobic conditions and also use lactic acid, causing nutrient loss.

Inoculation of both grains with Inoc1 resulted in silages dominated by the *Lactobacillus* genus, with much less diversity than CTRL and Inoc2. SG-Inoc2 silages could sustain more *Pantoea, Kosakonia*, and other *Enterobacteriaceae* that could potentially include pathogenic species of bacteria. Thus, the addition of Inoc1 may also contribute to the safety of SG silages.

A large number of other undesirable microorganisms may develop in silage when the pH is insufficiently reduced or when oxygen is available. Some species of *Bacillus, Clostridium, Listeria, Mycobacterium, Yersinia*, and *Salmonella* are associated with human or animal diseases^60^. Species of *Pseudomonas* and *Klebsiella* can produce biogenic amines, which are often linked to a decrease in the protein content and nutritional value of silage^61^. Among these genera, in our study, only *Bacillus, Clostridium*, and *Pseudomonas* were detected in both grains and *Mycobacterium* in CG samples (data not shown). However, the number of OTUs belonging to these genera was relatively low in all silages, reflecting the trace amounts of butyric acid present in the silages (Supplementary Table S2).

Increased richness and diversity, with a significant decrease in *Firmicutes* and an increase in *Proteobacteria* in the bacterial community, were also reported by Liu et al*.*^14^ in barley silages after prolonged aerobic exposure. Therefore, the reason for the changes in the bacterial community at 360 days in our study is unclear because the experimental conditions of anaerobiosis were constant throughout the period. In addition, studies analyzing bacterial communities are limited to shorter fermentation periods, making it difficult to correlate the observed results with those of previous studies.

Contrary to the reports of Dunière et al*.*^60^, in which silage could be stored for a long time due to acidification, changes in the final community of silages in both grains after 360 days break the premise that the bacterial community stabilizes after a given fermentation period. This suggests that even with a constant pH, changes in the microbial profile of grain silages may result in undesired changes, such as higher concentrations of NH_3_-N and propionic acid and lower LAB counts, in the fermentation profile of silages stored for a long time.

Notably, the epiphytic mycobiome profile of the grains before fermentation was different, resulting in different effects of the microbial inoculant on the fungal population of the ensiled materials. The predominance of *Ascomycota* and the presence of lower quantities of *Basidiomycota* and *Zygomycota* were also observed in wilted oats, corn, and their silages^31,62,63^. However, in our study, *Mucoromycota* was found in place of *Zygomycota*. Moreover, *Ascomycota* and *Basidiomycota* were the main phyla in the Purple prairie clover (*Dalea purpurea Vent*.)^16^.

The *Ascomycota* group is of particular relevance to humans as a source of medicinal compounds and food-making products, but also as pathogens of humans and plants. According to Romero et al*.*^31^, aerobic stability increases at a high relative abundance of unidentified *Ascomycota* species in corn silage, suggesting that this microorganism may have limited the growth of other microbes responsible for aerobic spoilage.

Although the predominance of *Ascomycota* was observed in all samples, the mycobiome profiles were different among the grains. The predominance of unidentified species of *Aspergillus* in the CG sample, and *Alternaria* and other unidentified fungi belonging to the order *Pleosparales* in SG samples before fermentation evidenced the high abundance of molds in pre-ensiled grains even without visible symptoms of fungal contamination. *Pleosporale* molds are typically associated with leaf spots on plants. Therefore, the presence of this genus was expected because many fungi inhabit the plant phyllosphere as they mature in the field and may still be present before absolute anaerobiosis is achieved^62^.

Most fungal microorganisms are strictly aerobic, and some *Penicillium*, *Fusarium*, and *Aspergillus* species remain after processing^63^. Therefore, in all mixed microbial populations of silages, certain species, such as *Aspergillus, Penicillium, Fusarium,* and *Monascus*^64^, are better adapted to reduced O_2_ tension, lower pH, and higher concentrations of carbon dioxide and organic acids^65^.

According to Gulbis et al*.*^66^, the most frequently isolated species from corn silage are fungi from the genera *Alternaria, Fusarium,* and *Penicillium.* In the present study, *Penicillium* and *Fusarium* spp. were present in low abundance during silage fermentation. Throughout the fermentation period, CG fermentation was dominated by *Aspergillus* molds, whereas *W. anomalus* yeast was predominant in the SG samples. Thus, the persistence and predominance of *Aspergillus* during the fermentation period in CG silages indicate that this genus is more tolerant to ensiling conditions than *Alternaria* and other *Pleosparal* molds present in SG before fermentation.

*Aspergillus* spp. is distributed worldwide and is among the most predominant species found in different silages in Brazil^67^. It requires high temperature and low water activity for growth and can survive under microaerophilic and acidic environments^68^. Moreover, mycotoxins produced by *Aspergillus* species are among the four major toxins found in corn silage^69^. The predominance of the *Aspergillus* genus in CG-Inoc1 silages since the beginning of fermentation suggests that the inoculant inhibited the growth of some yeasts, especially the *Wickerhamomyce*s species. However, the increased abundance of unidentified yeasts belonging to the order *Saccharomycetales* at 360 days was unexpected, and the reason for the same is unclear.

Several factors, including the amount of air ingressed during silage storage and the type of crop ensiled, are known to affect the yeast flora composition in silage^70^. As anaerobic conditions were maintained throughout the fermentation period, it is suggested that the different yeast community profiles among grains may be affected by crop type and other factors^71^. As discussed previously, the presence of some yeast in silage is associated with anaerobic fermentative loss and aerobic deterioration after the exposure of silage to air during animal feeding or damage in the sealing of the silo^70^. In addition to losses in silage quality, yeasts may also be opportunistic pathogens^72^ that reduce in vitro apparent neutral detergent fiber digestion^73^.

Yeasts such as the *Wickerhamomyce*s species found in this study belong to the order *Saccharomycetales* and are frequently associated with spoilage or processing of food and grain products. *Saccharomycetales* spp. are also predominant in the fungal core microbiome of small grain silages^34^. *Wickerhamomyce*s *anomalus*, formerly known as *Pichia anomala*, *Hansenula anomala,* or *Candida pelliculosa*, was assigned to the genus *Wickerhamomyces*^72^. It is a biotechnologically relevant yeast species that is present in diverse habitats^74^. The predominance of this species during the fermentation of SG silages is related to its ability to tolerate extreme environmental conditions, such as oxidative, salt, and osmotic stress, as well as pH and temperature shocks^75^. However, due to these characteristics, this microorganism can cause spoilage in several products, including silage and dairy products^76,77^.

According to Druvefors et al*.*^78^, *W. anomalus* is promising as an alternative to chemical fungicides in the storage of cereal grains because it produces antifungal metabolites derived from glycolysis, rather than from competition for nutrients or activity of cell wall lytic enzymes. Ethanol and ethyl acetate are also considered responsible for the antifungal activity of this species. In this context, the effects of *W. anomalus* on silage fermentation still need to be elucidated.

## Conclusions

The effects of inoculants on bacterial and fungal succession differed between the two grains. *Lactobacillus* and *Weissella* are the main bacteria involved in the fermentation of rehydrated silages of corn and sorghum grains. *Aspergillu*s spp. were predominant in rehydrated corn grain fermentation, whereas *W. anomalus* was the major fungal species in rehydrated sorghum grain silages. The inoculant containing *L.plantarum* and *P. acidipropionici* was more efficient in promoting a sharp growth of *Lactobacillus* and maintaining higher stability of the bacterial community during longer periods of storage in silages of both grains. Furthermore, it controlled the growth of *Wickerhamomyces* yeast until 90 days of fermentation in rehydrated corn grain silages. The bacterial and fungal communities of rehydrated corn and sorghum grain silages did not remain stable after 360 days of storage.

## Supplementary Information

Supplementary Information.

## Data Availability

The datasets used and/or analyzed during the current study are available in the Science data bank repository: https://doi.org/10.57760/sciencedb.02767.
