# Supplementary material for: Microbiome of rehydrated corn and sorghum grain silages treated with microbial inoculants in different fermentation periods
Source: Sci Rep. 2022 Oct 7;12:16864. doi: 10.1038/s41598-022-21461-4 (PMC9546842; doi:10.1038/s41598-022-21461-4)
Supplement: Supplementary file 1 — Supplementary Information. [file 41598_2022_21461_MOESM1_ESM.docx]

Microbiome of rehydrated corn and sorghum grain silages treated with microbial inoculants in different fermentation periods

Mariele Cristina Nascimento Agarussi, Odilon Gomes Pereira, Felipe Evangelista Pimentel, Camila Ferreira Azevedo, Vanessa Paula da Silva, Fabyano Fonseca e Silva

Supplementary Table S1. The deviance information criterion (DIC) of the models including the interaction effects.

| Grain | Population | Effects | DIC |
| --- | --- | --- | --- |
| Corn | Fungi | $u_{1},u_{2},u_{3},u_{4}$ | **1479.86** |
|  |  | $u_{1},u_{2},u_{3}$ | 1487.32 |
|  |  | $u_{1},u_{2},u_{4}$ | 1483.05 |
|  |  | $u_{1},u_{3},u_{4}$ | 1494.69 |
|  |  | $u_{1},u_{2}$ | 1497.64 |
|  |  | $u_{1},u_{3}$ | 1554.86 |
|  |  | $u_{1},u_{4}$ | 1496.43 |
|  |  | $u_{1}$ | 1556.49 |
|  | Bacteria | $u_{1},u_{2},u_{3},u_{4}$ | **6352.18** |
|  |  | $u_{1},u_{2},u_{3}$ | 6390.26 |
|  |  | $u_{1},u_{2},u_{4}$ | 6374.60 |
|  |  | $u_{1},u_{3},u_{4}$ | 6408.63 |
|  |  | $u_{1},u_{2}$ | 6437.47 |
|  |  | $u_{1},u_{3}$ | 6495.49 |
|  |  | $u_{1},u_{4}$ | 6417.77 |
|  |  | $u_{1}$ | 6501.96 |
| Sorghum | Fungi | $u_{1},u_{2},u_{3},u_{4}$ | **3888.82** |
|  |  | $u_{1},u_{2},u_{3}$ | 3913.70 |
|  |  | $u_{1},u_{2},u_{4}$ | 3894.30 |
|  |  | $u_{1},u_{3},u_{4}$ | 3923.21 |
|  |  | $u_{1},u_{2}$ | 3926.15 |
|  |  | $u_{1},u_{3}$ | 3992.67 |
|  |  | $u_{1},u_{4}$ | 3925.97 |
|  |  | $u_{1}$ | 3993.89 |
|  | Bacteria | $u_{1},u_{2},u_{3},u_{4}$ | 2365.95 |
|  |  | $u_{1},u_{2},u_{3}$ | 2365.99 |
|  |  | $u_{1},u_{2},u_{4}$ | **2361.21** |
|  |  | $u_{1},u_{3},u_{4}$ | 2402.27 |
|  |  | $u_{1},u_{2}$ | 2369.21 |
|  |  | $u_{1},u_{3}$ | 2448.96 |
|  |  | $u_{1},u_{4}$ | 2402.88 |
|  |  | $u_{1}$ | 2448.77 |

Supplementary Table S2. Average (with respective standard error of the mean and ANOVA based *P-value*) fermentation profile (% DM) and microbial population (log cfu/g of fresh weight) of rehydrated corn and sorghum grain silages throughout the fermentation period.

| Inoculant | Days | Grain | pH | WSC^1^ | Fermentation products | | | | | Microbial counts | | |
| --- | --- | --- | --- | --- | --- | --- | --- | --- | --- | --- | --- | --- |
|  |  |  |  |  | LA^2^ | AA^3^ | BA^4^ | PA^5^ | NH_3_-N | LAB^6^ | ENT^7^ | Fungi |
| CTRL | 3 | **CG** | 4.77 | 3.56 | 0.25 | 0.05 | 0.06 | 0.001 | 2.86 | 8.9 | 6.65 | 5.64 |
|  | 7 | **CG** | 4.32 | 3.33 | 0.35 | 0.06 | 0.08 | 0.001 | 3.19 | 8.71 | 5.28 | 5.37 |
|  | 21 | **CG** | 4.10 | 2.33 | 0.27 | 0.06 | 0.04 | 0.001 | 4.02 | 8.22 | 3.94 | 5.01 |
|  | 90 | **CG** | 3.87 | 2.05 | 0.76 | 0.11 | 0.12 | 0.002 | 4.63 | 6.74 | 2.61 | 2.91 |
|  | 360 | **CG** | 3.81 | 2.72 | 1.16 | 0.21 | 0.03 | 0.254 | 5.13 | 3.86 | 0 | 1.97 |
| Inoc1 | 3 | **CG** | 4.03 | 2.62 | 0.66 | 0.05 | 0.05 | 0.001 | 3.15 | 9.58 | 4.48 | 5.72 |
|  | 7 | **CG** | 3.9 | 2.88 | 0.90 | 0.05 | 0.05 | 0.001 | 3.42 | 8.38 | 3.81 | 5.51 |
|  | 21 | **CG** | 3.94 | 3.07 | 0.74 | 0.06 | 0.04 | 0.002 | 3.85 | 5.53 | 2.76 | 5.12 |
|  | 90 | **CG** | 3.93 | 2.50 | 0.85 | 0.05 | 0.07 | 0.005 | 4.30 | 4.91 | 0.92 | 4.72 |
|  | 360 | **CG** | 3.93 | 2.95 | 1.07 | 0.11 | 0.03 | 0.231 | 3.89 | 4.36 | 0 | 3.62 |
| Inoc2 | 3 | **CG** | 4.77 | 2.9 | 0.23 | 0.04 | 0.06 | 0 | 3.26 | 9.37 | 7.11 | 5.55 |
|  | 7 | **CG** | 4.14 | 2.4 | 0.67 | 0.13 | 0.15 | 0 | 4.21 | 9.70 | 3.24 | 4.79 |
|  | 21 | **CG** | 4.00 | 0.75 | 0.37 | 0.13 | 0.09 | 0.001 | 3.48 | 9.50 | 0 | 4.16 |
|  | 90 | **CG** | 3.98 | 2.33 | 0.95 | 0.27 | 0.19 | 0.001 | 5.62 | 4.72 | 1.03 | 2.03 |
|  | 360 | **CG** | 3.98 | 1.86 | 1.10 | 0.24 | 0.03 | 0.455 | 6.55 | 3.95 | 0 | 1.97 |
| CTRL | 3 | **SG** | 5.22 | 0.63 | 0.27 | 0.03 | 0.05 | 0.001 | 2.35 | 9 | 8.08 | 4.73 |
|  | 7 | **SG** | 4.73 | 0.68 | 0.35 | 0.04 | 0.07 | 0 | 2.59 | 9.18 | 6.19 | 5.97 |
|  | 21 | **SG** | 4.46 | 0.56 | 0.33 | 0.06 | 0.06 | 0.001 | 2.95 | 8.51 | 3.11 | 5.65 |
|  | 90 | **SG** | 4.44 | 0.75 | 0.42 | 0.08 | 0.12 | 0.001 | 3.65 | 8.55 | 1.76 | 4.09 |
|  | 360 | **SG** | 4.15 | 0.78 | 1.01 | 0.10 | 0.09 | 0.244 | 4.63 | 6.38 | 0 | 1.39 |
| Inoc1 | 3 | **SG** | 4.1 | 0.37 | 0.60 | 0.04 | 0.02 | 0.002 | 1.76 | 9.8 | 4.51 | 3.69 |
|  | 7 | **SG** | 4.03 | 0.48 | 0.77 | 0.05 | 0.03 | 0.002 | 2.12 | 9.08 | 3.92 | 3.88 |
|  | 21 | **SG** | 4.01 | 0.44 | 0.33 | 0.05 | 0.01 | 0.006 | 2.07 | 7.10 | 2.62 | 4.46 |
|  | 90 | **SG** | 4.00 | 0.68 | 0.76 | 0.04 | 0.04 | 0.003 | 2.59 | 6.40 | 0 | 3.46 |
|  | 360 | **SG** | 3.92 | 0.69 | 0.87 | 0.30 | 0.08 | 0.472 | 3.01 | 3.87 | 0 | 2.79 |
| Inoc2 | 3 | **SG** | 4.79 | 0.39 | 0.29 | 0.03 | 0.06 | 0 | 2.26 | 9.53 | 6.91 | 4.41 |
|  | 7 | **SG** | 4.52 | 0.49 | 0.40 | 0.07 | 0.09 | 0.001 | 2.86 | 9.46 | 3.99 | 4.19 |
|  | 21 | **SG** | 4.58 | 0.43 | 0.24 | 0.12 | 0.08 | 0.001 | 3.00 | 9.54 | 2.93 | 4.06 |
|  | 90 | **SG** | 4.46 | 0.47 | 0.30 | 0.39 | 0.15 | 0.004 | 3.64 | 8.22 | 0 | 3.36 |
|  | 360 | **SG** | 4.36 | 0.56 | 0.43 | 0.60 | 0.05 | 0.716 | 5.56 | 5.48 | 0 | 3.73 |
| SEM | | | 0.09 | 0.36 | 0.14 | 0.08 | 0.03 | 0.10 | 0.39 | 0.51 | 0.61 | 0.85 |
| *P-value* | I | | < 0.01 | < 0.01 | < 0.01 | < 0.01 | < 0.01 | 0.03 | < 0.01 | < 0.01 | < 0.01 | < 0.01 |
|  | G | | < 0.01 | < 0.01 | < 0.01 | 0.670 | < 0.01 | 0.108 | < 0.01 | < 0.01 | 0.716 | < 0.01 |
|  | P | | < 0.01 | < 0.01 | < 0.01 | < 0.01 | < 0.01 | < 0.01 | < 0.01 | < 0.01 | < 0.01 | < 0.01 |
|  | I × G | | < 0.01 | < 0.01 | < 0.01 | < 0.01 | < 0.01 | 0.489 | 0.178 | 0.235 | 0.10 | 0.134 |
|  | I × P | | < 0.01 | < 0.01 | < 0.01 | < 0.01 | < 0.01 | < 0.01 | < 0.01 | < 0.01 | < 0.01 | < 0.01 |
|  | G × P | | 0.0058 | < 0.01 | < 0.01 | < 0.01 | < 0.01 | 0.02 | 0.0059 | < 0.01 | < 0.027 | < 0.01 |
|  | I × P × G | | 0.001 | < 0.01 | < 0.01 | < 0.01 | <0.01 | 0.630 | 0.019 | < 0.01 | < 0.01 | 0.018 |

^1^Water soluble carbohydrate; ^2^Lactic acid; ^3^Acetic acid; ^4^Butyric acid; ^5^Propionic acid; ^6^Lactic acid bacteria; ^7^Enterobacteria. **CG:** Corn grain; **SG:** Sorghum grain. **CTRL:** non-inoculated; **Inoc1**: *Lactobacillus plantarum* and *Propionibacterium acidipropionici* and **Inoc2**: *Lactobacillus buchneri*. I: Inoculant effect; G: Grain effect; P: Fermentation period effect; I × G: Interaction inoculant × grain effect; I × P: Interaction inoculant × fermentation period effect; G × P: Interaction grain × fermentation period effect; I × P × G: Interaction inoculant × fermentation period × grain effect.

Supplementary Table S3. Bacterial abundance in rehydrated corn grain and theirs silages throughout 0, 3, 7, 21, 90 and 360 days of fermentation. CTRL: non-inoculated; Inoc1: Lactobacillus plantarum and Propionibacterium acidipropionici and Inoc2: Lactobacillus buchneri.

| Inoculant | CTRL | | | | | | Inoc1 | | | | | | Inoc2 | | | | | | |
| --- | --- | --- | --- | --- | --- | --- | --- | --- | --- | --- | --- | --- | --- | --- | --- | --- | --- | --- | --- |
| Fermentation period (days) | 0 | 3 | 7 | 21 | 90 | 360 | 0 | 3 | 7 | 21 | 90 | 360 | | 0 | 3 | 7 | 21 | 90 | 360 |
| *Acetobacterales, Acetobacteraceae, Craurococcus* | 3 | 0 | 0 | 0 | 0 | 0 | 0 | 0 | 0 | 0 | 0 | 0 | | 1 | 0 | 0 | 0 | 1 | 1 |
| *Acetobacterales, Acetobacteraceae, Roseomonas* | 7 | 0 | 0 | 0 | 0 | 2 | 0 | 0 | 0 | 0 | 0 | 0 | | 0 | 0 | 0 | 0 | 0 | 0 |
| *Actinomycetales, Actinomycetaceae, Actinomyces* | 0 | 0 | 0 | 0 | 0 | 0 | 0 | 0 | 0 | 0 | 0 | 0 | | 0 | 0 | 0 | 0 | 0 | 0 |
| *Bacillales, Bacillaceae, Bacillus* | 15 | 0 | 0 | 0 | 0 | 42 | 7 | 1 | 1 | 2 | 31 | 23 | | 3 | 763 | 932 | 980 | 964 | 214 |
| *Bacillales, Bacillaceae, Geobacillus* | 0 | 0 | 0 | 0 | 0 | 4 | 0 | 0 | 0 | 0 | 0 | 0 | | 6 | 0 | 0 | 0 | 0 | 0 |
| *Bacillales, Bacillaceae, Oceanobacillus* | 0 | 0 | 0 | 0 | 0 | 31 | 0 | 0 | 0 | 0 | 3 | 156 | | 18 | 0 | 0 | 0 | 0 | 0 |
| *Bacillales, Bacillaceae, Ureibacillus* | 0 | 0 | 0 | 0 | 0 | 0 | 0 | 0 | 0 | 0 | 2 | 2 | | 14 | 0 | 0 | 0 | 0 | 0 |
| *Bacillales, Bacillaceae, Virgibacillus* | 0 | 0 | 0 | 0 | 0 | 3 | 0 | 0 | 0 | 0 | 0 | 37 | | 0 | 0 | 0 | 0 | 0 | 1 |
| *Bacillales, Family XII, Exiguobacterium* | 0 | 0 | 0 | 0 | 0 | 0 | 1 | 0 | 0 | 0 | 0 | 0 | | 0 | 0 | 0 | 0 | 0 | 0 |
| *Bacillales, Paenibacillaceae, Brevibacillus* | 1 | 0 | 0 | 0 | 0 | 0 | 0 | 0 | 0 | 0 | 1 | 0 | | 0 | 3 | 0 | 1 | 3 | 2 |
| *Bacillales, Paenibacillaceae, Cohnella* | 1 | 0 | 0 | 0 | 0 | 0 | 0 | 0 | 0 | 0 | 1 | 1 | | 2 | 0 | 1 | 0 | 0 | 0 |
| *Bacillales, Paenibacillaceae, Paenibacillus* | 9 | 1 | 0 | 1 | 1 | 10 | 8 | 0 | 1 | 1 | 24 | 0 | | 10 | 1 | 0 | 0 | 0 | 2 |
| *Bacillales, Paenibacillaceae, Saccharibacillus* | 0 | 0 | 0 | 0 | 0 | 0 | 0 | 0 | 0 | 0 | 0 | 0 | | 0 | 0 | 0 | 0 | 0 | 0 |
| *Bacillales, Planococcaceae, Domibacillus* | 4 | 0 | 0 | 0 | 0 | 0 | 0 | 0 | 0 | 0 | 0 | 1 | | 1 | 0 | 0 | 0 | 0 | 0 |
| *Bacillales, Planococcaceae, Lysinibacillus* | 4 | 0 | 0 | 0 | 0 | 21 | 8 | 0 | 0 | 2 | 76 | 76 | | 213 | 181 | 9 | 4 | 5 | 78 |
| *Bacillales, Planococcaceae, Rummeliibacillus* | 0 | 0 | 0 | 0 | 0 | 2 | 0 | 0 | 0 | 0 | 1 | 6 | | 0 | 0 | 0 | 0 | 0 | 0 |
| *Bacillales, Planococcaceae, Solibacillus* | 3 | 0 | 0 | 0 | 0 | 2 | 1 | 0 | 0 | 0 | 4 | 2 | | 1 | 0 | 0 | 0 | 0 | 0 |
| *Bacillales, Staphylococcaceae, Staphylococcus* | 100 | 1 | 1 | 0 | 0 | 2 | 121 | 0 | 2 | 4 | 1 | 1 | | 9 | 0 | 0 | 0 | 0 | 0 |
| *Bacillales, Thermoactinomycetaceae, Shimazuella* | 1 | 0 | 0 | 0 | 0 | 0 | 0 | 0 | 0 | 0 | 0 | 0 | | 0 | 0 | 0 | 0 | 0 | 0 |
| *Bacteroidales, Dysgonomonadaceae, uncultured* | 2 | 0 | 0 | 0 | 0 | 0 | 0 | 0 | 0 | 0 | 1 | 0 | | 0 | 0 | 0 | 0 | 0 | 0 |
| *Bdellovibrionales, Bdellovibrionaceae, Bdellovibrio* | 0 | 0 | 0 | 0 | 0 | 0 | 2 | 0 | 0 | 0 | 0 | 0 | | 0 | 96 | 247 | 289 | 286 | 35 |
| *Chloroflexi, TK10, uncultured bacterium* | 2 | 0 | 0 | 0 | 0 | 0 | 0 | 0 | 0 | 0 | 0 | 0 | | 3 | 0 | 0 | 0 | 1 | 0 |
| *Chthoniobacterales, Chthoniobacteraceae, Candidatus Udaeobacter* | 1 | 0 | 0 | 0 | 0 | 0 | 0 | 0 | 0 | 0 | 0 | 1 | | 0 | 0 | 1 | 0 | 0 | 0 |
| *Clostridiales, Christensenellaceae, Christensenellaceae R-7 group* | 1 | 0 | 0 | 0 | 0 | 3 | 0 | 0 | 0 | 0 | 0 | 0 | | 2 | 0 | 0 | 0 | 0 | 0 |
| *Clostridiales, Clostridiaceae 1, Clostridium sensu stricto 1* | 0 | 0 | 0 | 0 | 0 | 4 | 1 | 0 | 0 | 0 | 9 | 4 | | 2 | 0 | 0 | 0 | 0 | 1 |
| *Clostridiales, Clostridiaceae 1, Clostridium sensu stricto 12* | 0 | 0 | 0 | 12 | 0 | 5 | 0 | 0 | 0 | 0 | 0 | 0 | | 18 | 0 | 0 | 0 | 0 | 0 |
| *Clostridiales, Clostridiaceae 1, Clostridium sensu stricto 13* | 1 | 0 | 0 | 0 | 0 | 0 | 0 | 0 | 0 | 0 | 0 | 0 | | 0 | 0 | 0 | 0 | 0 | 0 |
| *Clostridiales, Clostridiaceae 1, Clostridium sensu stricto 3* | 0 | 0 | 0 | 0 | 0 | 0 | 7 | 0 | 0 | 0 | 1 | 0 | | 0 | 0 | 0 | 0 | 0 | 0 |
| *Clostridiales, Clostridiaceae 1, Clostridium sensu stricto 8* | 0 | 0 | 0 | 0 | 0 | 9 | 0 | 0 | 0 | 0 | 0 | 0 | | 0 | 0 | 0 | 0 | 0 | 0 |
| *Clostridiales, Clostridiaceae 1, Clostridium sensu stricto 9* | 0 | 0 | 0 | 0 | 0 | 3 | 0 | 0 | 0 | 0 | 0 | 0 | | 0 | 0 | 0 | 0 | 0 | 0 |
| *Clostridiales, Clostridiaceae 2, Alkaliphilus* | 1 | 0 | 0 | 0 | 0 | 4 | 0 | 0 | 0 | 0 | 2 | 0 | | 0 | 0 | 0 | 0 | 0 | 0 |
| *Clostridiales, Family XI, Tissierella* | 0 | 0 | 0 | 0 | 0 | 2 | 0 | 0 | 0 | 0 | 1 | 0 | | 0 | 0 | 0 | 0 | 0 | 0 |
| *Clostridiales, Lachnospiraceae, Anaerocolumna* | 3 | 0 | 0 | 0 | 0 | 2 | 1 | 0 | 0 | 0 | 4 | 0 | | 0 | 84 | 51 | 16 | 25 | 19 |
| *Clostridiales, Lachnospiraceae, NA* | 1 | 0 | 0 | 0 | 0 | 0 | 6 | 0 | 0 | 0 | 10 | 0 | | 0 | 0 | 0 | 0 | 0 | 0 |
| *Clostridiales, Peptostreptococcaceae, Romboutsia* | 2 | 0 | 0 | 0 | 0 | 2 | 0 | 0 | 0 | 0 | 3 | 0 | | 0 | 0 | 0 | 0 | 0 | 0 |
| *Clostridiales, Peptostreptococcaceae, Sporacetigenium* | 0 | 0 | 0 | 0 | 0 | 0 | 0 | 0 | 0 | 0 | 3 | 0 | | 1 | 0 | 0 | 0 | 0 | 0 |
| *Corynebacteriales, Corynebacteriaceae, Corynebacterium 1* | 45 | 0 | 0 | 0 | 0 | 132 | 18 | 0 | 0 | 0 | 3 | 79 | | 4 | 0 | 0 | 0 | 0 | 0 |
| *Corynebacteriales, Corynebacteriaceae, Lawsonella* | 6 | 0 | 0 | 0 | 0 | 0 | 0 | 0 | 0 | 0 | 0 | 0 | | 3 | 0 | 0 | 0 | 0 | 0 |
| *Corynebacteriales, Mycobacteriaceae, Mycobacterium* | 11 | 1 | 0 | 0 | 0 | 0 | 21 | 1 | 2 | 2 | 0 | 0 | | 0 | 0 | 0 | 0 | 0 | 0 |
| *Corynebacteriales, Nocardiaceae, Gordonia* | 3 | 0 | 0 | 0 | 0 | 2 | 17 | 1 | 1 | 0 | 0 | 0 | | 0 | 0 | 0 | 0 | 0 | 3 |
| *Cytophagales, Spirosomaceae, Dyadobacter* | 2 | 0 | 0 | 0 | 0 | 0 | 3 | 0 | 0 | 0 | 0 | 0 | | 0 | 0 | 0 | 0 | 0 | 0 |
| *Cytophagales, Spirosomaceae, Larkinella* | 2 | 0 | 0 | 0 | 0 | 0 | 0 | 0 | 0 | 0 | 0 | 0 | | 0 | 0 | 0 | 0 | 0 | 0 |
| *Deinococcales, Deinococcaceae, Deinococcus* | 2 | 0 | 0 | 0 | 0 | 0 | 0 | 0 | 0 | 0 | 0 | 0 | | 0 | 0 | 0 | 0 | 0 | 0 |
| *Diplorickettsiales, Diplorickettsiaceae, uncultured* | 2 | 0 | 0 | 0 | 0 | 0 | 0 | 0 | 0 | 0 | 0 | 0 | | 0 | 0 | 0 | 0 | 0 | 0 |
| *Enterobacteriales, Enterobacteriaceae, Cronobacter* | 10 | 4 | 0 | 1 | 0 | 0 | 8 | 0 | 0 | 0 | 0 | 0 | | 1 | 0 | 0 | 0 | 0 | 1 |
| *Enterobacteriales, Enterobacteriaceae, Izhakiella* | 2 | 0 | 0 | 0 | 0 | 0 | 0 | 0 | 0 | 0 | 0 | 0 | | 2 | 0 | 0 | 0 | 0 | 0 |
| *Enterobacteriales, Enterobacteriaceae, Kosakonia* | 11 | 0 | 0 | 0 | 0 | 72 | 2 | 1 | 0 | 4 | 3 | 1 | | 1 | 0 | 0 | 0 | 0 | 0 |
| *Enterobacteriales, Enterobacteriaceae, NA* | 6 | 0 | 0 | 1 | 0 | 0 | 1 | 0 | 0 | 0 | 0 | 4 | | 4 | 0 | 0 | 0 | 0 | 0 |
| *Enterobacteriales, Enterobacteriaceae, Pantoea* | 152 | 33 | 10 | 4 | 0 | 41 | 92 | 2 | 1 | 17 | 13 | 3 | | 0 | 0 | 0 | 0 | 0 | 1 |
| *Enterobacteriales, Enterobacteriaceae, Sodalis* | 6 | 0 | 0 | 0 | 0 | 0 | 0 | 0 | 0 | 0 | 0 | 0 | | 3 | 0 | 0 | 0 | 0 | 0 |
| *Erysipelotrichales, Erysipelotrichaceae, Erysipelatoclostridium* | 2 | 0 | 0 | 0 | 0 | 0 | 0 | 0 | 0 | 0 | 0 | 1 | | 1 | 0 | 0 | 1 | 0 | 0 |
| *Erysipelotrichales, Erysipelotrichaceae, Turicibacter* | 3 | 0 | 0 | 0 | 0 | 0 | 1 | 0 | 0 | 0 | 2 | 0 | | 0 | 0 | 0 | 0 | 0 | 0 |
| *Flavobacteriales, Flavobacteriaceae, Flavobacterium* | 5 | 0 | 0 | 0 | 0 | 0 | 3 | 0 | 0 | 0 | 0 | 0 | | 1 | 0 | 0 | 0 | 0 | 0 |
| *Flavobacteriales, Flavobacteriaceae, NS3a marine group* | 0 | 0 | 0 | 0 | 0 | 5 | 0 | 0 | 0 | 0 | 0 | 0 | | 4 | 0 | 0 | 0 | 0 | 0 |
| *Flavobacteriales, Weeksellaceae, Chryseobacterium* | 69 | 0 | 1 | 0 | 0 | 0 | 7 | 0 | 0 | 0 | 0 | 0 | | 4 | 0 | 2 | 0 | 0 | 1 |
| *Flavobacteriales, Weeksellaceae, Elizabethkingia* | 0 | 0 | 0 | 0 | 0 | 0 | 0 | 0 | 0 | 0 | 0 | 0 | | 0 | 0 | 0 | 0 | 0 | 82 |
| *Flavobacteriales, Weeksellaceae, Empedobacter* | 0 | 0 | 0 | 0 | 0 | 0 | 1 | 0 | 0 | 0 | 1 | 0 | | 4 | 0 | 0 | 0 | 0 | 0 |
| *Frankiales, Geodermatophilaceae, Geodermatophilus* | 2 | 0 | 0 | 0 | 0 | 0 | 0 | 0 | 0 | 0 | 0 | 0 | | 0 | 0 | 0 | 0 | 0 | 4 |
| *Lactobacillales, Enterococcaceae, Enterococcus* | 2 | 36 | 10 | 31 | 2 | 14 | 3 | 0 | 0 | 0 | 0 | 5 | | 0 | 0 | 0 | 0 | 0 | 0 |
| *Lactobacillales, Lactobacillaceae, Lactobacillus* | 5 | 86 | 346 | 475 | 1223 | 424 | 4 | 1265 | 1260 | 1203 | 1042 | 644 | | 27 | 0 | 0 | 2 | 0 | 162 |
| *Lactobacillales, Lactobacillaceae, Pediococcus* | 0 | 43 | 17 | 23 | 1 | 7 | 2 | 2 | 0 | 0 | 0 | 1 | | 1 | 0 | 0 | 0 | 0 | 0 |
| *Lactobacillales, Leuconostocaceae, Leuconostoc* | 0 | 9 | 2 | 2 | 0 | 5 | 0 | 0 | 0 | 0 | 0 | 0 | | 13 | 0 | 0 | 0 | 0 | 130 |
| *Lactobacillales, Leuconostocaceae, Weissella* | 20 | 1047 | 900 | 756 | 90 | 137 | 3 | 21 | 23 | 13 | 10 | 12 | | 9 | 0 | 0 | 0 | 0 | 0 |
| *Lactobacillales, Streptococcaceae, Lactococcus* | 8 | 46 | 22 | 3 | 2 | 2 | 3 | 0 | 0 | 0 | 0 | 3 | | 115 | 1 | 0 | 0 | 1 | 12 |
| *Lactobacillales, Streptococcaceae, Streptococcus* | 1 | 0 | 0 | 0 | 0 | 0 | 0 | 0 | 0 | 0 | 1 | 0 | | 6 | 3 | 0 | 0 | 2 | 0 |
| *Micrococcales, Brevibacteriaceae, Brevibacterium* | 91 | 2 | 2 | 3 | 0 | 45 | 356 | 17 | 9 | 29 | 4 | 32 | | 0 | 0 | 0 | 3 | 0 | 0 |
| *Micrococcales, Cellulomonadaceae, Cellulomonas* | 0 | 0 | 0 | 0 | 0 | 0 | 0 | 0 | 0 | 0 | 0 | 0 | | 1 | 12 | 2 | 0 | 1 | 4 |
| *Micrococcales, Dermabacteraceae, Brachybacterium* | 15 | 0 | 0 | 0 | 0 | 1 | 86 | 1 | 0 | 0 | 0 | 5 | | 0 | 0 | 0 | 0 | 0 | 0 |
| *Micrococcales, Microbacteriaceae, Curtobacterium* | 2 | 0 | 0 | 0 | 1 | 0 | 2 | 0 | 0 | 0 | 0 | 0 | | 0 | 0 | 0 | 0 | 0 | 12 |
| *Micrococcales, Microbacteriaceae, NA* | 2 | 0 | 0 | 0 | 0 | 0 | 5 | 0 | 0 | 0 | 0 | 1 | | 1 | 0 | 0 | 0 | 0 | 0 |
| *Micrococcales, Micrococcaceae, Arthrobacter* | 3 | 0 | 0 | 0 | 0 | 138 | 6 | 0 | 0 | 0 | 0 | 168 | | 0 | 1 | 0 | 0 | 0 | 0 |
| *Micrococcales, Micrococcaceae, Glutamicibacter* | 0 | 0 | 0 | 0 | 0 | 0 | 1 | 0 | 0 | 0 | 0 | 0 | | 10 | 0 | 0 | 0 | 0 | 0 |
| *Micrococcales, Micrococcaceae, Kocuria* | 21 | 1 | 0 | 0 | 0 | 3 | 91 | 1 | 2 | 3 | 1 | 2 | | 3 | 0 | 0 | 0 | 0 | 0 |
| *Micrococcales, NA, NA* | 0 | 0 | 1 | 0 | 0 | 0 | 0 | 0 | 0 | 0 | 0 | 0 | | 0 | 0 | 0 | 0 | 0 | 1 |
| *Micrococcales, NA, NA* | 0 | 0 | 0 | 0 | 0 | 0 | 1 | 0 | 0 | 0 | 0 | 5 | | 1 | 0 | 0 | 0 | 0 | 0 |
| *Micrococcales, Promicromonosporaceae, Cellulosimicrobium* | 3 | 0 | 0 | 0 | 0 | 0 | 2 | 0 | 0 | 0 | 0 | 0 | | 0 | 61 | 3 | 3 | 7 | 0 |
| *Micromonosporales, Micromonosporaceae, NA* | 0 | 0 | 0 | 0 | 0 | 0 | 0 | 0 | 0 | 0 | 2 | 1 | | 1 | 0 | 0 | 0 | 0 | 0 |
| *Micromonosporales; Micromonosporaceae; Actinocatenispora* | 0 | 0 | 0 | 0 | 0 | 0 | 3 | 0 | 0 | 0 | 0 | 0 | | 2 | 0 | 0 | 0 | 0 | 0 |
| *Myxococcales, Polyangiaceae, NA* | 0 | 0 | 0 | 0 | 0 | 6 | 0 | 0 | 0 | 0 | 0 | 0 | | 10 | 0 | 0 | 0 | 0 | 1 |
| *NA* | 4 | 0 | 1 | 0 | 0 | 0 | 4 | 0 | 0 | 0 | 0 | 0 | | 0 | 1 | 0 | 0 | 0 | 0 |
| *Nitrosococcales, Methylophagaceae, Methylophaga* | 0 | 0 | 0 | 0 | 0 | 5 | 0 | 0 | 0 | 0 | 0 | 0 | | 41 | 0 | 0 | 0 | 2 | 0 |
| *Pasteurellales, Pasteurellaceae, uncultured* | 1 | 0 | 0 | 0 | 0 | 0 | 0 | 0 | 0 | 0 | 0 | 0 | | 1 | 0 | 0 | 0 | 0 | 0 |
| *Planctomycetales, Gimesiaceae, uncultured* | 0 | 0 | 0 | 0 | 0 | 3 | 0 | 0 | 0 | 0 | 0 | 0 | | 2 | 0 | 0 | 0 | 0 | 0 |
| *Pseudomonadales, Moraxellaceae, Acinetobacter* | 102 | 2 | 3 | 3 | 0 | 9 | 54 | 1 | 1 | 4 | 7 | 2 | | 3 | 0 | 1 | 0 | 0 | 3 |
| *Pseudomonadales, Moraxellaceae, Alkanindiges* | 0 | 0 | 0 | 0 | 0 | 0 | 0 | 0 | 0 | 0 | 0 | 0 | | 0 | 0 | 0 | 0 | 0 | 0 |
| *Pseudomonadales, Pseudomonadaceae, Pseudomonas* | 16 | 0 | 1 | 0 | 1 | 20 | 3 | 0 | 0 | 1 | 5 | 2 | | 5 | 0 | 0 | 0 | 0 | 0 |
| *Pseudonocardiales, Pseudonocardiaceae, Actinophytocola* | 1 | 0 | 0 | 0 | 0 | 0 | 15 | 0 | 1 | 0 | 2 | 0 | | 0 | 0 | 0 | 0 | 0 | 0 |
| *Pseudonocardiales, Pseudonocardiaceae, NA* | 3 | 0 | 0 | 0 | 0 | 0 | 1 | 0 | 0 | 0 | 0 | 0 | | 1 | 0 | 0 | 0 | 0 | 0 |
| *Pseudonocardiales, Pseudonocardiaceae, Pseudonocardia* | 2 | 0 | 0 | 0 | 0 | 0 | 0 | 0 | 1 | 1 | 0 | 0 | | 0 | 0 | 0 | 0 | 0 | 0 |
| *Rhizobiales, Beijerinckiaceae, Bosea* | 2 | 0 | 0 | 0 | 0 | 0 | 1 | 0 | 0 | 0 | 0 | 0 | | 0 | 3 | 0 | 0 | 0 | 0 |
| *Rhizobiales, Beijerinckiaceae, Camelimonas* | 0 | 0 | 0 | 0 | 0 | 0 | 2 | 0 | 0 | 2 | 0 | 0 | | 1 | 0 | 0 | 0 | 0 | 0 |
| *Rhizobiales, Beijerinckiaceae, Chelatococcus* | 1 | 0 | 0 | 0 | 0 | 0 | 2 | 0 | 0 | 0 | 0 | 0 | | 0 | 0 | 0 | 0 | 0 | 0 |
| *Rhizobiales, Beijerinckiaceae, Methylobacterium* | 0 | 0 | 0 | 0 | 0 | 0 | 1 | 0 | 0 | 0 | 0 | 0 | | 1 | 0 | 0 | 0 | 0 | 0 |
| *Rhizobiales, Devosiaceae, Devosia* | 3 | 0 | 0 | 0 | 0 | 0 | 1 | 0 | 0 | 0 | 0 | 0 | | 0 | 0 | 0 | 0 | 0 | 0 |
| *Rhizobiales, Kaistiaceae, Kaistia* | 21 | 0 | 0 | 0 | 0 | 0 | 3 | 0 | 0 | 1 | 0 | 0 | | 2 | 0 | 0 | 0 | 0 | 7 |
| *Rhizobiales, Rhizobiaceae, Allorhizobium-Neorhizobium-Pararhizobium-Rhizobium* | 10 | 0 | 0 | 0 | 0 | 0 | 8 | 0 | 1 | 2 | 0 | 0 | | 15 | 64 | 22 | 7 | 4 | 9 |
| *Rhizobiales, Rhizobiaceae, Aureimonas* | 2 | 0 | 0 | 0 | 0 | 0 | 0 | 0 | 0 | 0 | 0 | 0 | | 0 | 1 | 0 | 0 | 0 | 0 |
| *Rhizobiales, Rhizobiaceae, NA* | 0 | 0 | 0 | 0 | 0 | 0 | 1 | 0 | 0 | 0 | 0 | 0 | | 208 | 0 | 17 | 0 | 1 | 0 |
| *Rhizobiales, Rhizobiaceae, Ochrobactrum* | 21 | 0 | 0 | 0 | 0 | 0 | 30 | 1 | 1 | 2 | 1 | 0 | | 24 | 0 | 0 | 0 | 0 | 0 |
| *Rhizobiales, Rhizobiaceae, uncultured* | 0 | 0 | 0 | 0 | 0 | 12 | 0 | 0 | 0 | 0 | 0 | 17 | | 0 | 0 | 0 | 0 | 0 | 0 |
| *Rhizobiales, Xanthobacteraceae, NA* | 2 | 0 | 0 | 0 | 0 | 0 | 0 | 0 | 0 | 0 | 2 | 0 | | 22 | 0 | 1 | 0 | 0 | 7 |
| *Rhodobacterales, Rhodobacteraceae, NA* | 0 | 0 | 0 | 0 | 0 | 22 | 0 | 0 | 0 | 0 | 4 | 1 | | 188 | 2 | 17 | 0 | 5 | 425 |
| *Rhodobacterales, Rhodobacteraceae, Paracoccus* | 4 | 0 | 0 | 0 | 0 | 0 | 0 | 0 | 0 | 0 | 0 | 2 | | 7 | 0 | 0 | 0 | 0 | 0 |
| *Rickettsiales, Anaplasmataceae, Wolbachia* | 2 | 0 | 0 | 0 | 0 | 0 | 0 | 0 | 0 | 0 | 0 | 0 | | 0 | 0 | 0 | 0 | 0 | 0 |
| *Rickettsiales, Rickettsiaceae, Rickettsia* | 0 | 0 | 0 | 0 | 0 | 0 | 1 | 0 | 0 | 0 | 1 | 0 | | 2 | 0 | 0 | 0 | 0 | 0 |
| *Rubrobacterales, Rubrobacteriaceae, Rubrobacter* | 1 | 0 | 0 | 0 | 0 | 1 | 0 | 0 | 0 | 0 | 0 | 1 | | 1 | 0 | 0 | 0 | 0 | 0 |
| *Selenomonadales, Veillonellaceae, Anaerospora* | 0 | 0 | 0 | 0 | 0 | 0 | 0 | 0 | 0 | 0 | 1 | 0 | | 0 | 0 | 0 | 0 | 1 | 0 |
| *Solibacterales; Solibacteraceae (Subgroup 3); Bryobacter* | 1 | 0 | 0 | 0 | 0 | 0 | 1 | 0 | 0 | 0 | 0 | 0 | | 9 | 0 | 0 | 0 | 0 | 4 |
| *Solirubrobacterales, Solirubrobacteraceae, Conexibacter* | 0 | 0 | 0 | 0 | 0 | 3 | 0 | 0 | 0 | 0 | 0 | 0 | | 0 | 0 | 0 | 0 | 0 | 0 |
| *Solirubrobacterales, Solirubrobacteraceae, Patulibacter* | 1 | 0 | 0 | 0 | 0 | 0 | 1 | 0 | 1 | 0 | 0 | 0 | | 8 | 0 | 0 | 0 | 0 | 0 |
| *Sphingobacteriales, Sphingobacteriaceae, Arcticibacter* | 2 | 0 | 0 | 0 | 0 | 0 | 0 | 0 | 0 | 0 | 0 | 0 | | 0 | 8 | 3 | 2 | 1 | 0 |
| *Sphingobacteriales, Sphingobacteriaceae, Mucilaginibacter* | 0 | 0 | 0 | 0 | 0 | 0 | 0 | 0 | 0 | 0 | 0 | 0 | | 1 | 0 | 0 | 0 | 0 | 0 |
| *Sphingobacteriales, Sphingobacteriaceae, Olivibacter* | 0 | 0 | 0 | 0 | 0 | 0 | 6 | 0 | 0 | 0 | 0 | 0 | | 2 | 0 | 0 | 0 | 0 | 0 |
| *Sphingobacteriales, Sphingobacteriaceae, Pedobacter* | 0 | 0 | 0 | 0 | 0 | 0 | 2 | 0 | 0 | 0 | 0 | 0 | | 7 | 0 | 0 | 0 | 0 | 0 |
| *Sphingobacteriales, Sphingobacteriaceae, Solitalea* | 0 | 0 | 0 | 0 | 0 | 3 | 0 | 0 | 0 | 0 | 0 | 5 | | 5 | 0 | 0 | 0 | 0 | 0 |
| *Sphingobacteriales, Sphingobacteriaceae, Sphingobacterium* | 18 | 1 | 0 | 1 | 0 | 0 | 13 | 0 | 0 | 2 | 0 | 0 | | 16 | 0 | 1 | 0 | 0 | 1 |
| *Sphingomonadales, Sphingomonadaceae, Altererythrobacter* | 1 | 0 | 0 | 0 | 0 | 0 | 1 | 0 | 0 | 0 | 0 | 0 | | 0 | 4 | 4 | 3 | 2 | 0 |
| *Sphingomonadales, Sphingomonadaceae, Novosphingobium* | 5 | 0 | 0 | 1 | 0 | 0 | 2 | 0 | 0 | 0 | 0 | 0 | | 0 | 0 | 0 | 0 | 0 | 0 |
| *Sphingomonadales, Sphingomonadaceae, Sphingobium* | 7 | 0 | 0 | 0 | 0 | 0 | 1 | 0 | 0 | 1 | 0 | 0 | | 1 | 0 | 0 | 0 | 0 | 0 |
| *Sphingomonadales, Sphingomonadaceae, Sphingomonas* | 7 | 0 | 0 | 1 | 0 | 0 | 0 | 0 | 0 | 0 | 0 | 0 | | 0 | 0 | 0 | 0 | 0 | 0 |
| *Streptomycetales, Streptomycetaceae, Streptomyces* | 63 | 0 | 0 | 1 | 0 | 3 | 161 | 3 | 10 | 15 | 10 | 0 | | 9 | 0 | 0 | 0 | 0 | 0 |
| *Streptosporangiales, Nocardiopsaceae, Nocardiopsis* | 1 | 0 | 0 | 0 | 0 | 0 | 1 | 0 | 0 | 0 | 1 | 0 | | 1 | 0 | 0 | 0 | 0 | 0 |
| *Verrucomicrobiales, Rubritaleaceae, Luteolibacter* | 0 | 0 | 0 | 0 | 0 | 0 | 0 | 0 | 0 | 0 | 0 | 0 | | 5 | 7 | 1 | 0 | 2 | 7 |
| *Verrucomicrobiales, Verrucomicrobiaceae, Prosthecobacter* | 0 | 0 | 0 | 0 | 0 | 0 | 0 | 0 | 0 | 0 | 0 | 0 | | 0 | 0 | 0 | 0 | 0 | 0 |
| *Vibrionales, Vibrionaceae, Vibrio* | 0 | 0 | 0 | 0 | 0 | 6 | 0 | 0 | 0 | 0 | 2 | 0 | | 3 | 1 | 0 | 0 | 0 | 0 |
| *Xanthomonadales, Rhodanobacteraceae, Dokdonella* | 14 | 0 | 0 | 0 | 0 | 0 | 0 | 0 | 0 | 0 | 0 | 0 | | 0 | 0 | 0 | 0 | 0 | 0 |
| *Xanthomonadales, Rhodanobacteraceae, Dyella* | 54 | 0 | 0 | 0 | 0 | 0 | 1 | 0 | 0 | 0 | 0 | 0 | | 0 | 0 | 0 | 0 | 0 | 0 |
| *Xanthomonadales, Xanthomonadaceae, Luteimonas* | 4 | 0 | 0 | 0 | 0 | 0 | 1 | 0 | 0 | 1 | 0 | 0 | | 14 | 0 | 0 | 0 | 0 | 0 |
| *Xanthomonadales, Xanthomonadaceae, Pseudoxanthomonas* | 5 | 0 | 0 | 0 | 0 | 0 | 3 | 0 | 0 | 0 | 0 | 0 | | 2 | 0 | 0 | 0 | 0 | 0 |
| *Xanthomonadales, Xanthomonadaceae, Stenotrophomonas* | 21 | 1 | 1 | 2 | 0 | 0 | 9 | 0 | 0 | 1 | 6 | 0 | | 14 | 0 | 0 | 0 | 0 | 0 |

Supplementary Table S4. Bacterial abundance in rehydrated sorghum grain and theirs silages throughout 0, 3, 7, 21, 90 and 360 days of fermentation. CTRL: non-inoculated; Inoc1: *Lactobacillus plantarum* and *Propionibacterium acidipropionici* and Inoc2: *Lactobacillus buchneri*.

| Inoculant | CTRL | | | | | | Inoc1 | | | | | | | | | | | | | | | | | | Inoc2 | | | | | | | | | | | | | | | | | |
| --- | --- | --- | --- | --- | --- | --- | --- | --- | --- | --- | --- | --- | --- | --- | --- | --- | --- | --- | --- | --- | --- | --- | --- | --- | --- | --- | --- | --- | --- | --- | --- | --- | --- | --- | --- | --- | --- | --- | --- | --- | --- | --- |
| Fermentation period (days) | 0 | 3 | 7 | 21 | 90 | 360 | 0 | | | 3 | | | 7 | | | 21 | | | 90 | | | 360 | | | 0 | | | 3 | | | 7 | | | 21 | | | 90 | | | 360 | | |
| *Acetobacterales, Acetobacteraceae, Roseomonas* | 1 | 0 | 0 | 0 | 0 | 0 | 3 | | | 0 | | | 0 | | | 1 | | | 0 | | | 0 | | | 1 | | | 0 | | | 0 | | | 0 | | | 0 | | | 0 | | |
| *Bacillales, Bacillaceae, Bacillus* | 2 | 0 | 0 | 0 | 0 | 1 | 0 | | | 3 | | | 0 | | | 0 | | | 0 | | | 20 | | | 7 | | | 0 | | | 0 | | | 0 | | | 0 | | | 0 | | |
| *Bacillales, Paenibacillaceae, Paenibacillus* | 4 | 0 | 0 | 0 | 0 | 0 | 1 | | | 0 | | | 0 | | | 0 | | | 0 | | | 0 | | | 7 | | | 0 | | | 0 | | | 0 | | | 0 | | | 0 | | |
| *Bacillales, Paenibacillaceae, Saccharibacillus* | 3 | 0 | 0 | 0 | 0 | 0 | 2 | | | 0 | | | 0 | | | 0 | | | 0 | | | 0 | | | 3 | | | 0 | | | 0 | | | 0 | | | 0 | | | 0 | | |
| *Bacillales, Planococcaceae, Domibacillus* | 0 | 0 | 0 | 0 | 0 | 0 | 4 | | | 0 | | | 0 | | | 0 | | | 0 | | | 0 | | | 0 | | | 0 | | | 0 | | | 0 | | | 0 | | | 0 | | |
| *Bacillales, Planococcaceae, Lysinibacillus* | 0 | 0 | 0 | 0 | 0 | 2 | 4 | | | 0 | | | 0 | | | 0 | | | 0 | | | 4 | | | 6 | | | 0 | | | 0 | | | 0 | | | 0 | | | 0 | | |
| *Bacillales, Planococcaceae, Solibacillus* | 0 | 0 | 0 | 0 | 0 | 0 | 0 | | | 0 | | | 1 | | | 0 | | | 0 | | | 1 | | | 0 | | | 0 | | | 0 | | | 0 | | | 0 | | | 0 | | |
| *Bacillales, Staphylococcaceae, Staphylococcus* | 7 | 0 | 1 | 0 | 0 | 7 | 2 | | | 0 | | | 0 | | | 0 | | | 0 | | | 0 | | | 6 | | | 1 | | | 2 | | | 1 | | | 0 | | | 6 | | |
| *Betaproteobacteriales, Burkholderiaceae, Ambiguous taxa* | 3 | 0 | 0 | 0 | 0 | 0 | 3 | | | 0 | | | 0 | | | 0 | | | 0 | | | 0 | | | 1 | | | 0 | | | 0 | | | 0 | | | 0 | | | 0 | | |
| *Betaproteobacteriales, Burkholderiaceae, Comamonas* | 2 | 0 | 0 | 0 | 0 | 0 | 1 | | | 0 | | | 0 | | | 1 | | | 0 | | | 0 | | | 0 | | | 0 | | | 0 | | | 0 | | | 0 | | | 0 | | |
| *Betaproteobacteriales, Burkholderiaceae, Massilia* | 26 | 0 | 0 | 0 | 0 | 0 | 15 | | | 0 | | | 0 | | | 1 | | | 0 | | | 0 | | | 28 | | | 0 | | | 1 | | | 0 | | | 0 | | | 0 | | |
| *Betaproteobacteriales, Burkholderiaceae, NA* | 2 | 0 | 0 | 0 | 0 | 0 | 0 | | | 0 | | | 0 | | | 0 | | | 0 | | | 0 | | | 0 | | | 0 | | | 0 | | | 0 | | | 0 | | | 0 | | |
| *Caulobacterales, Caulobacteraceae, Brevundimonas* | 0 | 0 | 0 | 0 | 0 | 1 | 1 | | | 0 | | | 0 | | | 2 | | | 0 | | | 0 | | | 3 | | | 0 | | | 0 | | | 0 | | | 0 | | | 0 | | |
| *Chitinophagales, Chitinophagaceae, Filimonas* | 0 | 0 | 0 | 0 | 0 | 0 | 3 | | | 0 | | | 0 | | | 0 | | | 0 | | | 0 | | | 0 | | | 0 | | | 0 | | | 0 | | | 0 | | | 0 | | |
| *Clostridiales, Clostridiaceae 1, Clostridium sensu stricto 1* | 1 | 0 | 0 | 0 | 0 | 0 | 1 | | | 0 | | | 0 | | | 0 | | | 0 | | | 9 | | | 10 | | | 0 | | | 1 | | | 0 | | | 0 | | | 0 | | |
| *Clostridiales, Clostridiaceae 1, Clostridium sensu stricto 12* | 0 | 0 | 16 | 9 | 9 | 3 | 0 | | | 0 | | | 0 | | | 5 | | | 40 | | | 0 | | | 0 | | | 2 | | | 2 | | | 11 | | | 0 | | | 1 | | |
| *Clostridiales, Peptostreptococcaceae, Ambiguous_taxa* | 2 | 0 | 0 | 0 | 0 | 0 | | 0 | | | 0 | | | 0 | | | 0 | | | 0 | | | 0 | | | 0 | | | 0 | | | 0 | | | 0 | | | 0 | | | 0 | |
| *Clostridiales, Peptostreptococcaceae, Romboutsia* | 1 | 0 | 0 | 0 | 0 | 0 | | 0 | | | 0 | | | 0 | | | 0 | | | 0 | | | 3 | | | 3 | | | 0 | | | 0 | | | 0 | | | 0 | | | 0 | |
| *Corynebacteriales, Corynebacteriaceae, Corynebacterium 1* | 2 | 0 | 1 | 0 | 0 | 4 | | 3 | | | 0 | | | 0 | | | 0 | | | 0 | | | 14 | | | 23 | | | 0 | | | 0 | | | 0 | | | 0 | | | 9 | |
| *Corynebacteriales, Nocardiaceae, Rhodococcus* | 0 | 0 | 0 | 0 | 0 | 0 | | 0 | | | 0 | | | 0 | | | 0 | | | 0 | | | 0 | | | 3 | | | 0 | | | 0 | | | 0 | | | 0 | | | 0 | |
| *Deinococcales, Deinococcaceae, Deinococcus* | 0 | 0 | 0 | 0 | 0 | 0 | | 0 | | | 0 | | | 0 | | | 0 | | | 0 | | | 0 | | | 4 | | | 0 | | | 0 | | | 0 | | | 0 | | | 0 | |
| *Enterobacteriales, Enterobacteriaceae, Cronobacter* | 16 | 41 | 35 | 1 | 18 | 7 | | 5 | | | 25 | | | 37 | | | 43 | | | 6 | | | 0 | | | 1 | | | 24 | | | 9 | | | 29 | | | 4 | | | 3 | |
| *Enterobacteriales, Enterobacteriaceae, Kosakonia* | 33 | 80 | 117 | 55 | 34 | 26 | | 1 | | | 22 | | | 16 | | | 31 | | | 4 | | | 2 | | | 9 | | | 216 | | | 244 | | | 66 | | | 84 | | | 202 | |
| *Enterobacteriales, Enterobacteriaceae, Pantoea* | 627 | 677 | 461 | 17 | 83 | 46 | | 1038 | | | 142 | | | 138 | | | 127 | | | 35 | | | 5 | | | 680 | | | 192 | | | 66 | | | 101 | | | 25 | | | 28 | |
| *Erysipelotrichales, Erysipelotrichaceae, Turicibacter* | 5 | 0 | 0 | 0 | 0 | 0 | | 0 | | | 0 | | | 0 | | | 0 | | | 0 | | | 5 | | | 11 | | | 0 | | | 1 | | | 0 | | | 0 | | | 0 | |
| *Flavobacteriales, Weeksellaceae, Apibacter* | 0 | 0 | 0 | 0 | 0 | 0 | | 0 | | | 0 | | | 1 | | | 0 | | | 0 | | | 0 | | | 1 | | | 0 | | | 0 | | | 0 | | | 0 | | | 0 | |
| *Flavobacteriales, Weeksellaceae, Chryseobacterium* | 18 | 0 | 0 | 0 | 0 | 0 | | 7 | | | 1 | | | 0 | | | 2 | | | 0 | | | 0 | | | 18 | | | 0 | | | 0 | | | 0 | | | 0 | | | 0 | |
| *Flavobacteriales, Weeksellaceae, Elizabethkingia* | 6 | 0 | 0 | 0 | 0 | 0 | | 3 | | | 1 | | | 0 | | | 0 | | | 0 | | | 0 | | | 15 | | | 0 | | | 0 | | | 0 | | | 0 | | | 0 | |
| *Frankiales, Geodermatophilaceae, Geodermatophilus* | 1 | 0 | 0 | 0 | 0 | 0 | | 0 | | | 0 | | | 0 | | | 0 | | | 0 | | | 0 | | | 1 | | | 0 | | | 0 | | | 0 | | | 0 | | | 0 | |
| *Kineosporiales, Kineosporiaceae, Quadrisphaera* | 6 | 0 | 0 | 0 | 0 | 0 | | 0 | | | 0 | | | 0 | | | 0 | | | 0 | | | 0 | | | 7 | | | 0 | | | 0 | | | 0 | | | 0 | | | 0 | |
| *Lactobacillales, Aerococcaceae, Aerococcus* | 0 | 0 | 0 | 0 | 0 | 0 | | 1 | | | 0 | | | 0 | | | 0 | | | 0 | | | 0 | | | 4 | | | 0 | | | 0 | | | 0 | | | 0 | | | 0 | |
| *Lactobacillales, Carnobacteriaceae, Desemzia* | 0 | 0 | 0 | 0 | 0 | 0 | | 1 | | | 0 | | | 0 | | | 0 | | | 0 | | | 0 | | | 2 | | | 0 | | | 0 | | | 0 | | | 0 | | | 0 | |
| *Lactobacillales, Carnobacteriaceae, uncultured* | 0 | 0 | 0 | 0 | 0 | 0 | | | 0 | | | 0 | | | 0 | | | 0 | | | 0 | | | 0 | | | 2 | | | 0 | | | 0 | | | 0 | | | 0 | | | 0 |
| *Lactobacillales, Enterococcaceae, Enterococcus* | 1 | 58 | 72 | 36 | 38 | 33 | | | 1 | | | 13 | | | 11 | | | 27 | | | 6 | | | 0 | | | 4 | | | 66 | | | 42 | | | 109 | | | 39 | | | 53 |
| *Lactobacillales, Lactobacillaceae, Lactobacillus* | 2 | 31 | 117 | 877 | 796 | 419 | | | 1 | | | 756 | | | 718 | | | 521 | | | 1120 | | | 1217 | | | 9 | | | 436 | | | 669 | | | 346 | | | 810 | | | 429 |
| *Lactobacillales, Lactobacillaceae, Pediococcus* | 1 | 24 | 29 | 38 | 30 | 5 | | | 0 | | | 21 | | | 20 | | | 17 | | | 1 | | | 0 | | | 0 | | | 59 | | | 48 | | | 52 | | | 33 | | | 33 |
| *Lactobacillales, Leuconostocaceae, Leuconostoc* | 0 | 19 | 13 | 9 | 8 | 22 | | | 0 | | | 43 | | | 42 | | | 70 | | | 12 | | | 3 | | | 0 | | | 31 | | | 11 | | | 6 | | | 34 | | | 30 |
| *Lactobacillales, Leuconostocaceae, Weissella* | 9 | 283 | 373 | 267 | 288 | 725 | | | 1 | | | 246 | | | 257 | | | 437 | | | 88 | | | 11 | | | 35 | | | 256 | | | 203 | | | 537 | | | 279 | | | 504 |
| *Lactobacillales, Streptococcaceae, Lactococcus* | 1 | 95 | 80 | 11 | 16 | 12 | | | 2 | | | 38 | | | 41 | | | 24 | | | 2 | | | 0 | | | 9 | | | 31 | | | 15 | | | 56 | | | 9 | | | 9 |
| *Micrococcales, Dermatophilaceae, NA* | 1 | 0 | 0 | 0 | 0 | 0 | | | 2 | | | 0 | | | 0 | | | 0 | | | 0 | | | 0 | | | 1 | | | 0 | | | 0 | | | 0 | | | 0 | | | 0 |
| *Micrococcales, Microbacteriaceae, Curtobacterium* | 1 | 0 | 0 | 0 | 0 | 0 | | | 14 | | | 0 | | | 0 | | | 0 | | | 0 | | | 0 | | | 9 | | | 0 | | | 0 | | | 0 | | | 0 | | | 0 |
| *Micrococcales, Microbacteriaceae, NA* | 0 | 0 | 0 | 0 | 0 | 0 | | | 0 | | | 0 | | | 0 | | | 0 | | | 0 | | | 0 | | | 2 | | | 0 | | | 0 | | | 0 | | | 0 | | | 0 |
| *Micrococcales, Micrococcaceae, Arthrobacter* | 0 | 0 | 0 | 0 | 0 | 1 | | | 0 | | | 0 | | | 0 | | | 0 | | | 0 | | | 12 | | | 0 | | | 0 | | | 0 | | | 0 | | | 0 | | | 3 |
| *Micrococcales, Micrococcaceae, Glutamicibacter* | 1 | 0 | 0 | 0 | 0 | 0 | | | 1 | | | 0 | | | 0 | | | 0 | | | 0 | | | 0 | | | 0 | | | 0 | | | 0 | | | 0 | | | 0 | | | 0 |
| *Micrococcales, Micrococcaceae, Kocuria* | 1 | 0 | 0 | 0 | 0 | 0 | | | 3 | | | 0 | | | 0 | | | 0 | | | 0 | | | 0 | | | 9 | | | 0 | | | 0 | | | 0 | | | 0 | | | 0 |
| *Nostocales, Chroococcidiopsaceae, Chroococcidiopsis PCC 7203* | 3 | 0 | 0 | 0 | 0 | 0 | | | 0 | | | 0 | | | 0 | | | 0 | | | 0 | | | 0 | | | 0 | | | 0 | | | 0 | | | 0 | | | 0 | | | 0 |
| *Nostocales, Phormidiaceae, NA* | 3 | 0 | 0 | 0 | 0 | 0 | | | 0 | | | 0 | | | 0 | | | 0 | | | 0 | | | 0 | | | 0 | | | 0 | | | 0 | | | 0 | | | 0 | | | 0 |
| *Pseudomonadales, Moraxellaceae, Acinetobacter* | 159 | 5 | 4 | 0 | 0 | 1 | | | 25 | | | 2 | | | 5 | | | 4 | | | 0 | | | 3 | | | 73 | | | 1 | | | 1 | | | 3 | | | 2 | | | 0 |
| *Pseudomonadales, Pseudomonadaceae, Pseudomonas* | 116 | 1 | 0 | 0 | 0 | 3 | | | 9 | | | 0 | | | 0 | | | 1 | | | 1 | | | 1 | | | 39 | | | 0 | | | 0 | | | 1 | | | 1 | | | 0 |
| *Rhizobiales, Beijerinckiaceae, Methylobacterium* | 3 | 0 | 0 | 0 | 0 | 0 | | | 1 | | | 0 | | | 0 | | | 0 | | | 0 | | | 1 | | | 7 | | | 0 | | | 0 | | | 0 | | | 0 | | | 0 |
| *Rhizobiales, Rhizobiaceae, Allorhizobium-Neorhizobium-Pararhizobium-Rhizobium* | 4 | 1 | 0 | 0 | 0 | 0 | | | 1 | | | 0 | | | 0 | | | 0 | | | 0 | | | 0 | | | 4 | | | 0 | | | 0 | | | 0 | | | 0 | | | 1 |
| *Rhizobiales, Rhizobiaceae, Aureimonas* | 13 | 0 | 1 | 0 | 0 | 0 | | | 7 | | | 1 | | | 1 | | | 0 | | | 1 | | | 0 | | | 18 | | | 0 | | | 0 | | | 0 | | | 0 | | | 0 |
| *Rhizobiales, Rhizobiaceae, Ochrobactrum* | 4 | 1 | 0 | 0 | 0 | 0 | | | 1 | | | 1 | | | 0 | | | 0 | | | 0 | | | 0 | | | 1 | | | 1 | | | 0 | | | 0 | | | 0 | | | 0 |
| *Rhodobacterales, Rhodobacteraceae, Paracoccus* | 1 | 0 | 0 | 0 | 0 | 1 | | | 2 | | | 0 | | | 0 | | | 0 | | | 0 | | | 1 | | | 22 | | | 0 | | | 0 | | | 0 | | | 0 | | | 0 |
| *Rhodobacterales, Rhodobacteraceae, Rubellimicrobium* | 0 | 0 | 0 | 0 | 0 | 0 | | | 0 | | | 0 | | | 0 | | | 0 | | | 0 | | | 0 | | | 2 | | | 0 | | | 0 | | | 0 | | | 0 | | | 0 |
| *Sphingobacteriales, Sphingobacteriaceae, Sphingobacterium* | 21 | 0 | 0 | 0 | 0 | 0 | | | 0 | | | 0 | | | 0 | | | 0 | | | 1 | | | 0 | | | 8 | | | 0 | | | 0 | | | 0 | | | 0 | | | 0 |
| *Sphingomonadales, Sphingomonadaceae, Novosphingobium* | 0 | 0 | 0 | 0 | 0 | 0 | | | 1 | | | 0 | | | 0 | | | 0 | | | 0 | | | 0 | | | 2 | | | 0 | | | 0 | | | 0 | | | 0 | | | 0 |
| *Sphingomonadales, Sphingomonadaceae, Sphingomonas* | 80 | 3 | 0 | 0 | 1 | 0 | | | 102 | | | 0 | | | 0 | | | 3 | | | 2 | | | 0 | | | 91 | | | 2 | | | 0 | | | 0 | | | 0 | | | 0 |
| *Xanthomonadales, Xanthomonadaceae, Stenotrophomonas* | 44 | 2 | 0 | 0 | 0 | 0 | | | 23 | | | 1 | | | 32 | | | 0 | | | 0 | | | 0 | | | 14 | | | 0 | | | 1 | | | 0 | | | 0 | | | 0 |
| *Xanthomonadales, Xanthomonadaceae, Xanthomonas* | 0 | 0 | 0 | 0 | 0 | 0 | | | 0 | | | 0 | | | 0 | | | 0 | | | 0 | | | 0 | | | 3 | | | 0 | | | 0 | | | 0 | | | 0 | | | 0 |


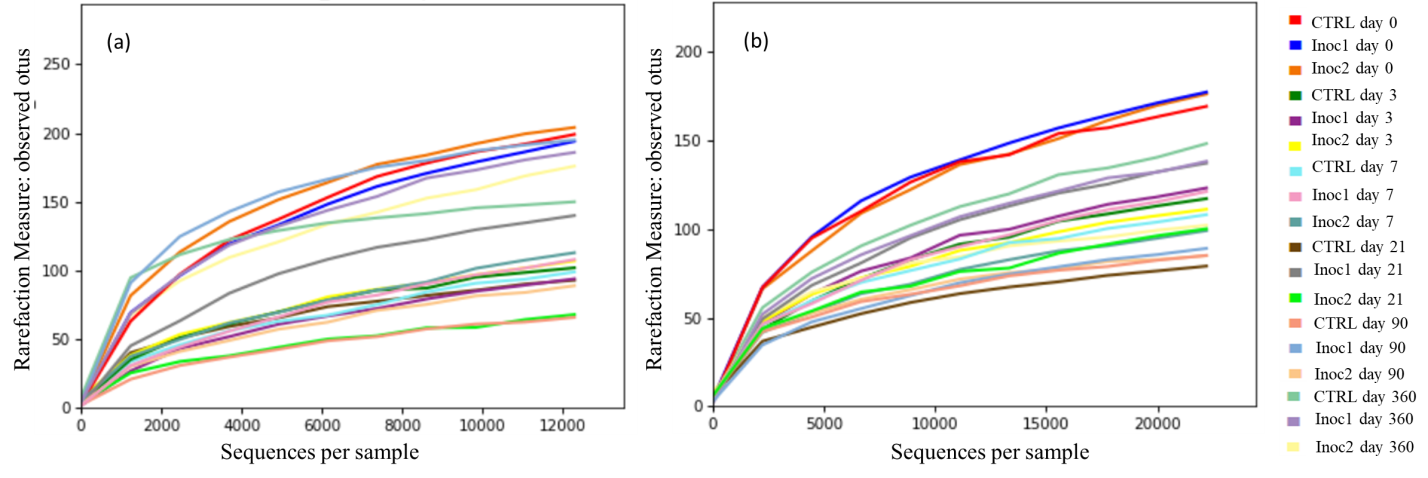


Supplementary Figure S1. Rarefaction curves showing the sampling effort and number of bacterial OTUs observed in rehydrated corn (a) and sorghum (b) grain silages after 0, 3, 7, 21, 90 and 360 d of fermentation. CTRL: non-inoculated; Inoc1: *Lactobacillus plantarum* and *Propionibacterium acidipropionici* and Inoc2: *Lactobacillus buchneri*.


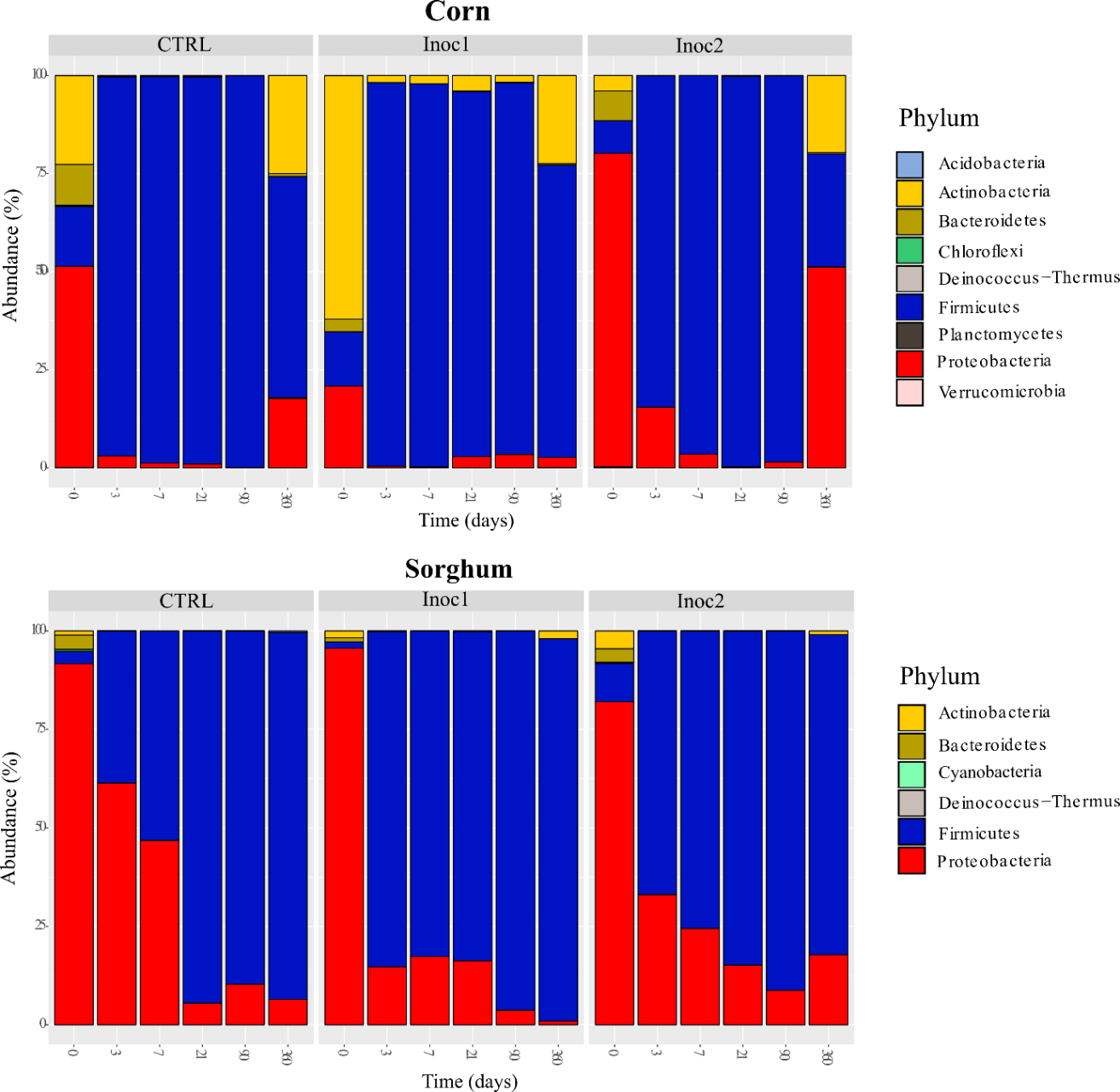


Supplementary Figure S2. Phyla taxonomic profiles of bacterial communities of rehydrated corn and sorghum grain silages after 0, 3, 7, 21, 90 and 360 days of fermentation. CTRL: non-inoculated; Inoc1: *Lactobacillus plantarum* and *Propionibacterium acidipropionici* and Inoc2: *Lactobacillus buchneri*.

Supplementary Table S5. Fungal abundance in rehydrated corn grain silages throughout 0, 3, 7, 21, 90 and 360 days of fermentation. CTRL: non-inoculated; Inoc1: *Lactobacillus plantarum* and *Propionibacterium acidipropionici* and Inoc2: *Lactobacillus buchneri***.**

| Inoculant | CTRL | | | | | | | Inoc1 | | | | | | | | | | | | | | | | | | | | | | Inoc2 | | | | | | | | | | | | | | | | | | | |
| --- | --- | --- | --- | --- | --- | --- | --- | --- | --- | --- | --- | --- | --- | --- | --- | --- | --- | --- | --- | --- | --- | --- | --- | --- | --- | --- | --- | --- | --- | --- | --- | --- | --- | --- | --- | --- | --- | --- | --- | --- | --- | --- | --- | --- | --- | --- | --- | --- | --- |
| Fermentation period (days) | 0 | 3 | 7 | | 21 | 90 | 360 | 0 | | | 3 | | | 7 | | | 21 | | | 90 | | | 360 | | | 0 | | | | | 3 | | | | 7 | | | | 21 | | | | 90 | | | | 360 | | |
| *Ascomycota, Classe* | 0 | 0 | 0 | | 0 | 0 | 25 | 0 | | | 0 | | | 0 | | | 0 | | | 0 | | | 0 | | | 0 | | | | | 0 | | | | 0 | | | | 0 | | | | 0 | | | | 1 | | |
| *Ascomycota, unidentified* | 0 | 0 | 0 | | 0 | 0 | 0 | 0 | | | 0 | | | 0 | | | 0 | | | 0 | | | 0 | | | 0 | | | | | 0 | | | | 0 | | | | 0 | | | | 0 | | | | 1 | | |
| *Basidiomycota, NA* | 0 | 0 | 0 | | 0 | 0 | 0 | 1 | | | 0 | | | 0 | | | 0 | | | 0 | | | 0 | | | 0 | | | | | 0 | | | | 0 | | | | 0 | | | | 0 | | | | 3 | | |
| *Cantharellales, unidentified* | 0 | 0 | 0 | | 0 | 0 | 0 | 0 | | | 0 | | | 0 | | | 0 | | | 0 | | | 0 | | | 0 | | | | | 0 | | | | 0 | | | | 0 | | | | 0 | | | | 2 | | |
| *Chaetothyriales, Chaetothyriales Incertae sedis, Sarcinomyces* | 1 | 0 | 0 | | 0 | 0 | 0 | 0 | | | 0 | | | 0 | | | 0 | | | 0 | | | 0 | | | 0 | | | | | 0 | | | | 0 | | | | 0 | | | | 0 | | | | 0 | | |
| *Chaetothyriales, Herpotrichiellaceae, Exophiala* | 0 | 0 | 0 | | 0 | 0 | 0 | 1 | | | 1 | | | 0 | | | 0 | | | 0 | | | 0 | | | 0 | | | | | 0 | | | | 0 | | | | 0 | | | | 0 | | | | 0 | | |
| *Corticiales, Vuilleminiaceae, Vuilleminia* | 0 | 0 | 0 | | 0 | 0 | 0 | 0 | | | 0 | | | 0 | | | 0 | | | 0 | | | 0 | | | 0 | | | | | 0 | | | | 0 | | | | 0 | | | | 0 | | | | 1 | | |
| *Cystofilobasidiales, NA* | 0 | 0 | 0 | | 0 | 0 | 48 | 0 | | | 0 | | | 0 | | | 0 | | | 0 | | | 0 | | | 0 | | | | | 0 | | | | 0 | | | | 0 | | | | 1 | | | | 4 | | |
| *Diaporthales, Diaporthaceae, NA* | 3 | 0 | 1 | | 0 | 0 | 0 | 1 | | | 3 | | | 0 | | | 0 | | | 0 | | | 0 | | | 2 | | | | | 1 | | | | 0 | | | | 0 | | | | 0 | | | | 0 | | |
| *Eurotiales, Aspergillaceae, Aspergillus* | 1470 | 897 | 456 | | 866 | 2616 | 2300 | 2534 | | | 2784 | | | 2653 | | | 2372 | | | 2814 | | | 377 | | | 1458 | | | | | 392 | | | | 1527 | | | | 2516 | | | | 2739 | | | | 2171 | | |
| *Eurotiales, Aspergillaceae, Monascus* | 0 | 0 | 0 | | 0 | 0 | 166 | 0 | | | 0 | | | 0 | | | 0 | | | 0 | | | 20 | | | 0 | | | | | 0 | | | | 0 | | | | 0 | | | | 0 | | | | 193 | | |
| *Eurotiales, Aspergillaceae, Penicillium* | 14 | 0 | 0 | | 0 | 0 | 0 | 8 | | | 1 | | | 0 | | | 0 | | | 0 | | | 0 | | | 2 | | | | | 0 | | | | 0 | | | | 0 | | | | 0 | | | | 2 | | |
| *Eurotiales, Aspergillaceae, Xeromyces* | 7 | 2 | 0 | | 1 | 1 | 0 | 0 | | | 0 | | | 0 | | | 0 | | | 1 | | | 0 | | | | 19 | | | | | 1 | | | | 1 | | | | 1 | | | | 6 | | | | 1 | |
| *Eurotiomycetes, NA* | 8 | 0 | 0 | | 0 | 0 | 62 | 5 | | | 1 | | | 0 | | | 0 | | | 0 | | | 0 | | | | 0 | | | | | 0 | | | | 0 | | | | 0 | | | | 1 | | | | 0 | |
| *Filobasidiales, Filobasidiaceae, Naganishia* | 677 | 6 | 1 | | 1 | 0 | 3 | 42 | | | 3 | | | 3 | | | 0 | | | 0 | | | 0 | | | | 360 | | | | | 1 | | | | 4 | | | | 0 | | | | 0 | | | | 0 | |
| *Filobasidiales, NA* | 0 | 0 | 0 | | 0 | 0 | 28 | 0 | | | 0 | | | 0 | | | 0 | | | 0 | | | 0 | | | | 0 | | | | | 0 | | | | 0 | | | | 0 | | | | 0 | | | | 4 | |
| *Hymenochaetales, Hymenochaetales Incertae sedis, Resinicium* | 1 | 0 | 0 | | 0 | 0 | 0 | 0 | | | 0 | | | 0 | | | 0 | | | 0 | | | 0 | | | | 0 | | | | | 0 | | | | 0 | | | | 0 | | | | 1 | | | | 0 | |
| *Hypocreales, Nectriaceae, Fusarium* | 15 | 1 | 0 | | 0 | 0 | 0 | 8 | | | 1 | | | 0 | | | 0 | | | 0 | | | 0 | | | | 14 | | | | | 3 | | | | 1 | | | | 0 | | | | 0 | | | | 0 | |
| *Mucorales, Mucoraceae, Mucor* | 16 | 30 | 44 | | 5 | 0 | 17 | 29 | | | 11 | | | 10 | | | 1 | | | 0 | | | 0 | | | | 138 | | | | | 565 | | | | 111 | | | | 16 | | | | 0 | | | | 5 | |
| *Mucorales, Rhizopodaceae, Rhizopus* | 483 | 14 | 11 | | 1 | 0 | 0 | 124 | | | 0 | | | 0 | | | 4 | | | 0 | | | 0 | | | | 428 | | | | | 63 | | | | 6 | | | | 6 | | | | 0 | | | | 3 | |
| *Orbiliales, unidentified,* | 4 | 0 | 0 | | 0 | 0 | 0 | 0 | | | 0 | | | 0 | | | 0 | | | 0 | | | 0 | | | | 0 | | | | | 0 | | | | 0 | | | | 0 | | | | 0 | | | | 0 | |
| *Pezizales, Ascodesmidaceae, Cephaliophora* | 0 | 0 | 0 | | 0 | 0 | 0 | 0 | | | 0 | | | 0 | | | 0 | | | 0 | | | 0 | | | | 2 | | | | | 0 | | | | 0 | | | | 0 | | | | 0 | | | | 0 | |
| *Pleosporales, NA* | 0 | 0 | 0 | | 0 | 0 | 0 | 1 | | | 0 | | | 0 | | | 0 | | | 0 | | | 0 | | | | 0 | | | | | 0 | | | | 0 | | | | 0 | | | | 0 | | | | 0 | |
| *Pleosporales, Pleosporaceae, Alternaria* | 1 | 0 | 0 | | 0 | 0 | 0 | 0 | | | 0 | | | 0 | | | 0 | | | 0 | | | 0 | | | | 0 | | | | | 0 | | | | 0 | | | | 0 | | | | 0 | | | | 0 | |
| *Pleosporales, unidentified* | 1 | 0 | 0 | | 0 | 0 | 0 | 0 | | | 0 | | | 0 | | | 0 | | | 0 | | | 0 | | | | 0 | | | | | 0 | | | | 0 | | | | 0 | | | | 1 | | | | 0 | |
| *Polyporales, Fomitopsidaceae, Dacryobolus* | 0 | 0 | 0 | | 0 | 1 | 0 | 0 | | | 0 | | | 0 | | | 0 | | | 0 | | | 0 | | | | 0 | | | | | 0 | | | | 0 | | | | 0 | | | | 0 | | | | 0 | |
| *Polyporales, Hyphodermataceae, Hyphoderma* | 0 | 0 | | 0 | 0 | 0 | 0 | | 0 | | | 0 | | | 0 | | | 0 | | | 0 | | | 0 | | | | 0 | | | | | 0 | | | | 0 | | | | 0 | | | | 5 | | | 0 | |
| *Polyporales, NA* | 0 | 0 | | 0 | 0 | 0 | 0 | | 0 | | | 0 | | | 0 | | | 0 | | | 0 | | | 0 | | | | 0 | | | | | 0 | | | | 0 | | | | 0 | | | | 1 | | | 0 | |
| *Polyporales, unidentified* | 0 | 0 | | 0 | 0 | 1 | 0 | | 0 | | | 0 | | | 0 | | | 0 | | | 0 | | | 0 | | | | 0 | | | | | 0 | | | | 0 | | | | 0 | | | | 0 | | | 0 | |
| *Polyporales, Xenasmataceae, Phlebiella* | 0 | 0 | | 0 | 0 | 0 | 0 | | 0 | | | 0 | | | 0 | | | 0 | | | 0 | | | 0 | | | | 0 | | | | | 0 | | | | 0 | | | | 0 | | | | 3 | | | 2 | |
| *Russulales, Peniophoraceae, Peniophora* | 0 | 1 | | 0 | 0 | 3 | 0 | | 0 | | | 0 | | | 0 | | | 0 | | | 0 | | | 0 | | | | 0 | | | | | 0 | | | | 0 | | | | 0 | | | | 0 | | | 0 | |
| *Saccharomycetales, Debaryomycetaceae, Debaryomyces* | 17 | 1 | | 0 | 0 | 0 | 0 | | 10 | | | 0 | | | 0 | | | 0 | | | 0 | | | 0 | | | | 11 | | | | | 1 | | | | 0 | | | | 0 | | | | 0 | | | 1 | |
| *Saccharomycetales, NA* | 12 | 4 | | 1 | 1 | 0 | 88 | | 5 | | | 2 | | | 4 | | | 1 | | | 0 | | | 2461 | | | | 5 | | | | | 0 | | | | 1 | | | | 4 | | | | 0 | | | 0 | |
| *Saccharomycetales, Phaffomycetaceae, Cyberlindnera* | 0 | 0 | | 1 | 0 | 0 | 0 | | 0 | | | 1 | | | 0 | | | 0 | | | 0 | | | 0 | | | | 0 | | | | | 0 | | | | 0 | | | | 0 | | | | 0 | | | 0 | |
| *Saccharomycetales, Phaffomycetaceae, Wickerhamomyces* | 39 | 1703 | | 2345 | 1985 | 240 | 89 | | 7 | | | 45 | | | 175 | | | 475 | | | 48 | | | 4 | | | | 81 | | | | | 1819 | | | | 1206 | | | | 311 | | | | 98 | | | 211 | |
| *Saccharomycetales, Saccharomycetales Incertae sedis, Candida* | 56 | 199 | | 3 | 3 | 0 | 16 | | 64 | | | 8 | | | 13 | | | 8 | | | 0 | | | 1 | | | | 245 | | | | | 13 | | | | 2 | | | | 6 | | | | 5 | | | 246 | |
| *Saccharomycetales, Trichomonascaceae, Blastobotrys* | 0 | 0 | | 0 | 0 | 0 | 1 | | 0 | | | 0 | | | 0 | | | 0 | | | 0 | | | 0 | | | | 0 | | | | | 0 | | | | 0 | | | | 0 | | | | 0 | | | 3 | |
| *Saccharomycetales, Trichomonascaceae, Zygoascus* | 0 | 1 | | 0 | 0 | 0 | 0 | | 0 | | | 0 | | | 0 | | | 0 | | | 0 | | | 0 | | | | 0 | | | | | 0 | | | | 2 | | | | 0 | | | | 0 | | | 0 | |
| *Sordariomycetes, unidentified* | 0 | 0 | | 0 | 0 | 0 | 0 | | 0 | | | 0 | | | 2 | | | 0 | | | 0 | | | 0 | | | | 0 | | | | | 0 | | | | 0 | | | | 0 | | | | 0 | | | 0 | |
| *Sporidiobolales, Sporidiobolaceae, Rhodotorula* | 14 | 0 | | 0 | 0 | 0 | 0 | | 5 | | | 0 | | | 0 | | | 0 | | | 0 | | | 0 | | | | 63 | | | | | 1 | | | | 0 | | | | 0 | | | | 0 | | | 5 | |
| *Trechisporales, Hydnodontaceae, Trechispora* | 0 | 0 | | 0 | 0 | 0 | 0 | | | 0 | | | 0 | | | 0 | | | 0 | | | 0 | | | 0 | | | | 0 | | | | | 0 | | | | 0 | | | | 0 | | | | 0 | | | 1 |
| *Tremellales, Cryptococcaceae, Kwoniella* | 8 | 3 | | 0 | 0 | 0 | 0 | | | 11 | | | 1 | | | 2 | | | 2 | | | 0 | | | 0 | | | | 15 | | | | | 0 | | | | 0 | | | | 3 | | | | 0 | | | 0 |
| *Wallemiales, Wallemiaceae, Wallemia* | 0 | 0 | | 0 | 0 | 0 | 0 | | | 0 | | | 0 | | | 0 | | | 0 | | | 0 | | | 0 | | | | 1 | | | | | 0 | | | | 0 | | | | 0 | | | | 0 | | | 0 |
| *Xylariales, Xylariales Incertae sedis, Phialemoniopsis* | 2 | 0 | | 0 | 0 | 0 | 0 | | | 1 | | | 0 | | | 0 | | | 0 | | | 0 | | | 0 | | | | 3 | | | | | 0 | | | | 0 | | | | 0 | | | | 1 | | | 0 |

Supplementary Table S6. Fungal abundance in rehydrated sorghum grain silages throughout 0, 3, 7, 21, 90 and 360 days of fermentation. CTRL: non-inculated; Inoc1: *Lactobacillus plantarum* and *Propionibacterium acidipropionici* and Inoc2: *Lactobacillus buchneri*.

| Inoculant | CTRL | | | | | | | | | | | | | | | | | | | | | | Inoc1 | | | | | | | | | | | | | | | | | | | | | | | | Inoc2 | | | | | | | | | | | | | | | | | | | | | |  |  |
| --- | --- | --- | --- | --- | --- | --- | --- | --- | --- | --- | --- | --- | --- | --- | --- | --- | --- | --- | --- | --- | --- | --- | --- | --- | --- | --- | --- | --- | --- | --- | --- | --- | --- | --- | --- | --- | --- | --- | --- | --- | --- | --- | --- | --- | --- | --- | --- | --- | --- | --- | --- | --- | --- | --- | --- | --- | --- | --- | --- | --- | --- | --- | --- | --- | --- | --- | --- | --- | --- | --- |
| Fermentation period (days) | 0 | | | 3 | | | | 7 | | | | 21 | | | | 90 | | | 360 | | | | 0 | | | | 3 | | | | 7 | | | | 21 | | | | 90 | | | 360 | | | | | 0 | | | | 3 | | | | | 7 | | | | 21 | | | | | 90 | | | |  |  |
| *Eurotiales, Aspergillaceae, Aspergillus* | 12 | | | 2 | | | | 2 | | | | 15 | | | | 5 | | | 1 | | | | 5 | | | | 11 | | | | 3 | | | | 22 | | | | 85 | | | 332 | | | | | 6 | | | | 1 | | | | | 7 | | | | 2 | | | | | 0 | | | |  |  |
| *Eurotiales, Aspergillaceae, Aspergillus, Aspergillus flavus* | 1 | | | 0 | | | | 0 | | | | 0 | | | | 0 | | | 0 | | | | 0 | | | | 0 | | | | 1 | | | | 0 | | | | 0 | | | 0 | | | | | 0 | | | | 0 | | | | | 0 | | | | 0 | | | | | 0 | | | |  |  |
| *Hypocreales, Nectriaceae, Fusarium* | 1 | | | 0 | | | | 0 | | | | 0 | | | | 0 | | | 1 | | | | 0 | | | | 0 | | | | 0 | | | | 0 | | | | 0 | | | 28 | | | | | 0 | | | | 0 | | | | | 0 | | | | 0 | | | | | 0 | | | |  |  |
| *Pleosporales, Phaeosphaeriaceae, Setophoma, Setophoma sacchari* | 0 | | | 0 | | | | 0 | | | | 0 | | | | 0 | | | 0 | | | | 0 | | | | 0 | | | | 0 | | | | 3 | | | | 0 | | | 0 | | | | | 0 | | | | 0 | | | | | 0 | | | | 0 | | | | | 0 | | | |  |  |
| *Pleosporales, Pleosporaceae, Bipolaris* | 0 | | | 0 | | | | 0 | | | | 0 | | | | 0 | | | 0 | | | | 2 | | | | 0 | | | | 0 | | | | 0 | | | | 0 | | | 0 | | | | | 2 | | | | 0 | | | | | 0 | | | | 0 | | | | | 0 | | | |  |  |
| *Sporidiobolales, Sporidiobolaceae, Rhodotorula* | 0 | | | 0 | | | | 0 | | | | 0 | | | | 0 | | | 0 | | | | 6 | | | | 1 | | | | 1 | | | | 1 | | | | 0 | | | 0 | | | | | 0 | | | | 0 | | | | | 0 | | | | 0 | | | | | 0 | | | |  |  |
| *Agaricales, Schizophyllaceae, Schizophyllum, Schizophyllum commune* | 1 | | | 0 | | | | 0 | | | | 0 | | | | 392 | | | 108 | | | | 0 | | | | 0 | | | | 0 | | | | 0 | | | | 1 | | | 0 | | | | | 0 | | | | 0 | | | | | 0 | | | | 0 | | | | | 0 | | | |  |  |
| *Agaricales, Strophariaceae, Psilocybe* | 0 | | | 0 | | | | 0 | | | | 0 | | | | 0 | | | 0 | | | | 0 | | | | 2 | | | | 0 | | | | 0 | | | | 0 | | | 0 | | | | | 0 | | | | 0 | | | | | 0 | | | | 0 | | | | | 0 | | | |  |  |
| *Agaricomycetes, NA* | 0 | | | 0 | | | | 0 | | | | 0 | | | | 0 | | | 0 | | | | 0 | | | | 0 | | | | 0 | | | | 0 | | | | 0 | | | 183 | | | | | 0 | | | | 0 | | | | | 0 | | | | 0 | | | | | 0 | | | |  |  |
| *Agaricomycetes, unidentified* | 0 | | | 0 | | | | 0 | | | | 0 | | | | 0 | | | 0 | | | | 0 | | | | 0 | | | | 0 | | | | 0 | | | | 5 | | | 110 | | | | | 0 | | | | 0 | | | | | 0 | | | | 0 | | | | | 0 | | | |  |  |
| *Agaricostilbales, Agaricostilbaceae, Sterigmatomyces, Sterigmatomyces halophilus* | 0 | | | 0 | | | | 0 | | | | 0 | | | | 0 | | | 0 | | | | 0 | | | | 0 | | | | 0 | | | | 0 | | | | 0 | | | 40 | | | | | 0 | | | | 0 | | | | | 0 | | | | 0 | | | | | 0 | | | |  |  |
| *Ascomycota, NA* | 2 | | | 0 | | | | 0 | | | | 4 | | | | 0 | | | 0 | | | | 2 | | | | 5 | | | | 0 | | | | 1 | | | | 0 | | | 0 | | | | | 4 | | | | 6 | | | | | 0 | | | | 0 | | | | | 0 | | | |  |  |
| *Ascomycota, unidentified* | 2 | | | 0 | | | | 0 | | | | 0 | | | | 0 | | | 0 | | | | 0 | | | | 0 | | | | 0 | | | | 0 | | | | 0 | | | 0 | | | | | 0 | | | | 0 | | | | | 0 | | | | 0 | | | | | 0 | | | |  |  |
| *Auriculariales, Exidiaceae, Heterochaete, NA* | 0 | | | 0 | | | | 0 | | | | 0 | | | | 0 | | | 0 | | | | 0 | | | | 0 | | | | 0 | | | | 1 | | | | 30 | | | 0 | | | | | 0 | | | | 0 | | | | | 0 | | | | 0 | | | | | 0 | | | |  |  |
| *Auriculariales, NA* | 0 | | | 0 | | | | 0 | | | | 0 | | | | 0 | | | 0 | | | | 0 | | | | 0 | | | | 0 | | | | 2 | | | | 0 | | | 0 | | | | | 0 | | | | 0 | | | | | 0 | | | | 0 | | | | | 0 | | | |  |  |
| *Basidiomycota, NA* | 9 | | | 1 | | | | 0 | | | | 1 | | | | 0 | | | 0 | | | | 4 | | | | 4 | | | | 2 | | | | 3 | | | | 0 | | | 0 | | | | | 8 | | | | 0 | | | | | 2 | | | | 0 | | | | | 0 | | | |  |  |
| *Cantharellales, Ceratobasidiaceae, Ceratobasidium* | 0 | | | 0 | | | | 0 | | | | 0 | | | | 0 | | | 0 | | | | 0 | | | | 0 | | | | 0 | | | | 0 | | | | 3 | | | 0 | | | | | 0 | | | | 0 | | | | | 0 | | | | 0 | | | | | 0 | | | |  |  |
| *Capnodiales, Mycosphaerellaceae, NA* | 2 | | | 0 | | | | 0 | | | | 1 | | | | 0 | | | 0 | | | | 0 | | | | 0 | | | | 0 | | | | 0 | | | | 0 | | | 0 | | | | | 2 | | | | 0 | | | | | 0 | | | | 0 | | | | | 0 | | | |  |  |
| *Capnodiales, NA* | 0 | | | 0 | | | | 0 | | | | 0 | | | | 0 | | | 0 | | | | 0 | | | | 0 | | | | 0 | | | | 0 | | | | 0 | | | 4 | | | | | 0 | | | | 0 | | | | | 0 | | | | 0 | | | | | 0 | | | |  |  |
| *Chaetothyriales, Chaetothyriales Incertae sedis, Sarcinomyces* | 3 | | | 0 | | | | 0 | | | | 0 | | | | 0 | | | 0 | | | | 0 | | | | 0 | | | | 0 | | | | 0 | | | | 0 | | | 0 | | | | | 0 | | | | 0 | | | | | 0 | | | | 0 | | | | | 0 | | | |  |  |
| *Chaetothyriales, Chaetothyriales Incertae sedis, Strelitziana, Strelitziana eucalypti* | 1 | | | 0 | | | | 0 | | | | 1 | | | | 0 | | | 0 | | | | 0 | | | | 0 | | | | 0 | | | | 0 | | | | 0 | | | 0 | | | | | 0 | | | | 1 | | | | | 0 | | | | 0 | | | | | 0 | | | |  |  |
| *Corticiales, NA* | 0 | | | 0 | | | | 0 | | | | 0 | | | | 0 | | | 85 | | | | 0 | | | | 0 | | | | 0 | | | | 0 | | | | 0 | | | 0 | | | | | 0 | | | | 0 | | | | | 0 | | | | 0 | | | | | 0 | | | |  |  |
| *Cystobasidiomycetes Incertae sedis, Symmetrosporaceae, Symmetrospora* | 1 | | | 0 | | | | 0 | | | | 1 | | | | 0 | | | 0 | | | | 1 | | | | 0 | | | | 2 | | | | 0 | | | | 0 | | | 0 | | | | | 2 | | | | 1 | | | | | 0 | | | | 0 | | | | | 0 | | | |  |  |
| *Cystofilobasidiales, NA* | 0 | | | 0 | | | | 0 | | | | 0 | | | | 1 | | | 0 | | | | 0 | | | | 0 | | | | 0 | | | | 11 | | | | 6 | | | 0 | | | | 0 | | | | 0 | | | | | 0 | | | | 0 | | | | | 0 | | | | |  |  |
| *Diaporthales, unidentified* | 0 | | | 1 | | | | 0 | | | | 0 | | | | 0 | | | 0 | | | | 0 | | | | 1 | | | | 1 | | | | 0 | | | | 0 | | | 0 | | | | 0 | | | | 0 | | | | | 0 | | | | 0 | | | | | 0 | | | | |  |  |
| *Dothideomycetes, Pleosporales, unidentified* | 0 | | | 0 | | | | 0 | | | | 0 | | | | 0 | | | 0 | | | | 0 | | | | 0 | | | | 0 | | | | 0 | | | | 0 | | | 0 | | | | 1 | | | | 0 | | | | | 1 | | | | 0 | | | | | 0 | | | | |  |  |
| *Dothideomycetes, unidentified* | 0 | | | 0 | | | | 0 | | | | 0 | | | | 0 | | | 0 | | | | 0 | | | | 0 | | | | 0 | | | | 0 | | | | 6 | | | 0 | | | | 0 | | | | 0 | | | | | 0 | | | | 0 | | | | | 0 | | | | |  |  |
| *Eurotiales, Aspergillaceae, Aspergillus* | 0 | | | 0 | | | | 0 | | | | 0 | | | | 0 | | | 1 | | | | 0 | | | | 0 | | | | 0 | | | | 0 | | | | 0 | | | 3 | | | | 0 | | | | 0 | | | | | 0 | | | | 0 | | | | | 0 | | | | |  |  |
| *Eurotiales, Aspergillaceae, Aspergillus, Aspergillus flavus* | 3 | | | 0 | | | | 0 | | | | 1 | | | | 2 | | | 1 | | | | 5 | | | | 0 | | | | 2 | | | | 2 | | | | 1 | | | 5 | | | | 3 | | | | 0 | | | | | 0 | | | | 0 | | | | | 0 | | | | |  |  |
| *Eurotiales, Aspergillaceae, Monascus, Monascus purpureus* | 0 | | | 0 | | | | 0 | | | | 0 | | | | 0 | | | 547 | | | | 0 | | | | 0 | | | | 0 | | | | 0 | | | | 1 | | | 154 | | | | 0 | | | | 0 | | | | | 0 | | | | 0 | | | | | 0 | | | | |  |  |
| *Eurotiales, Aspergillaceae, Penicillium* | 0 | | | 0 | | | | 0 | | | | 0 | | | | 0 | | | 56 | | | | 0 | | | | 0 | | | | 0 | | | | 0 | | | | 0 | | | 15 | | | | 0 | | | | 0 | | | | | 0 | | | | 0 | | | | | 0 | | | | |  |  |
| *Eurotiales, Aspergillaceae, Xeromyces, Xeromyces bisporus* | 1 | | | 0 | | | | 0 | | | | 0 | | | | 0 | | | 0 | | | | 0 | | | | 0 | | | | 0 | | | | 2 | | | | 0 | | | 0 | | | | 0 | | | | 0 | | | | | 0 | | | | 0 | | | | | 0 | | | | |  |  |
| *Eurotiomycetes, NA* | 0 | | | 0 | | | | 0 | | | | 0 | | | | 0 | | | 210 | | | | 0 | | | | 0 | | | | 0 | | | | 1 | | | | 3 | | | 456 | | | | 0 | | | | 0 | | | | | 0 | | | | 0 | | | | | 0 | | | | |  |  |
| *Filobasidiales, Filobasidiaceae, Naganishia* | 35 | | | 1 | | | | 0 | | | | 1 | | | | 1 | | | 61 | | | | 451 | | | | 28 | | | | 30 | | | | 18 | | | | 0 | | | 1 | | | | 33 | | | | 3 | | | | | 62 | | | | 0 | | | | | 0 | | | | |  |  |
| *Hymenochaetales, Hymenochaetales Incertae sedis, Resinicium* | 0 | | | 0 | | | | 0 | | | | 0 | | | | 0 | | | 0 | | | | 0 | | | | 0 | | | | 0 | | | | 0 | | | | 5 | | | 0 | | | | 0 | | | | 0 | | | | | 0 | | | | 0 | | | | | 0 | | | | |  |  |
| *Hymenochaetales, Hymenochaetales Incertae sedis, Resinicium, Resinicium saccharicola* | 0 | | | 0 | | | | 0 | | | | 0 | | | | 0 | | | 0 | | | | 0 | | | | 0 | | | | 0 | | | | 1 | | | | 1 | | | 0 | | | | 0 | | | | 0 | | | | | 0 | | | | 0 | | | | | 0 | | | | |  |  |
| *Hypocreales, Clavicipitaceae, Claviceps* | 0 | | | 0 | | | | 0 | | | | 0 | | | | 0 | | | 0 | | | | 0 | | | | 0 | | | | 1 | | | | 1 | | | | 0 | | | 0 | | | | 0 | | | | 0 | | | | | 0 | | | | 0 | | | | | 0 | | | | |  |  |
| *Hypocreales, Nectriaceae, Fusarium* | 1 | | | 0 | | | | 0 | | | | 0 | | | | 0 | | | 0 | | | | 0 | | | | 1 | | | | 1 | | | | 0 | | | | 0 | | | 0 | | | | 2 | | | | 0 | | | | | 0 | | | | 0 | | | | | 0 | | | | |  |  |
| *Mucorales, Mucoraceae, Mucor, Mucor circinelloides* | 15 | | | 2 | | | | 2 | | | | 0 | | | | 0 | | | 0 | | | | 3 | | | | 97 | | | | 33 | | | | 38 | | | | 2 | | | 0 | | | | 0 | | | | 1 | | | | | 0 | | | | 0 | | | | | 0 | | | | |  |  |
| *Mucorales, Rhizopodaceae, Rhizopus, Rhizopus arrhizus* | 1 | | | 0 | | | | 0 | | | | 1 | | | | 0 | | | 0 | | | | 1 | | | | 0 | | | | 0 | | | | 2 | | | | 0 | | | 0 | | | | 0 | | | | 0 | | | | | 0 | | | | 0 | | | | | 0 | | | | |  |  |
| *Pleosporales, Massarinaceae, Saccharicola* | 0 | | | 8 | | | | 0 | | | | 0 | | | | 0 | | | 0 | | | | 0 | | | | 0 | | | | 0 | | | | 0 | | | | 0 | | | 0 | | | | 0 | | | | 0 | | | | | 16 | | | | 0 | | | | | 0 | | | | |  |  |
| *Pleosporales, Massarinaceae, Stagonospora* | 0 | | | 0 | | | | 0 | | | | 0 | | | | 0 | | | 0 | | | | 0 | | | | 0 | | | | 0 | | | | 0 | | | | 8 | | | 0 | | | | 0 | | | | 0 | | | | | 0 | | | | 0 | | | | | 0 | | | | |  |  |
| *Pleosporales, Periconiaceae, Periconia* | 2 | | | 0 | | | | 0 | | | | 0 | | | | 0 | | | 0 | | | | 0 | | | | 0 | | | | 0 | | | | 0 | | | | 0 | | | 0 | | | | 0 | | | | 0 | | | | | 0 | | | | 0 | | | | | 0 | | | | |  |  |
| *Pleosporales, Phaeosphaeriaceae, NA* | 1 | | | 0 | | | | 0 | | | | 0 | | | | 0 | | | 0 | | | | 1 | | | | 0 | | | | 0 | | | | 0 | | | | 0 | | | 0 | | | | 0 | | | | 0 | | | | | 0 | | | | 0 | | | | | 0 | | | | |  |  |
| *Pleosporales, Pleosporaceae, Alternaria* | 687 | | | 88 | | | | 53 | | | | 59 | | | | 7 | | | 54 | | | | 467 | | | | 125 | | | | 163 | | | | 375 | | | | 77 | | | 8 | | | | 362 | | | | 106 | | | | | 331 | | | | 5 | | | | | 1 | | | | |  |  |
| *Pleosporales, Pleosporaceae, Bipolaris* | 10 | | | 7 | | | | 1 | | | | 4 | | | | 0 | | | 0 | | | | 6 | | | | 2 | | | | 3 | | | | 17 | | | | 3 | | | 0 | | | | 12 | | | | 2 | | | | | 5 | | | | 0 | | | | | 0 | | | | |  |  |
| *Pleosporales, Pleosporaceae, Exserohilum* | | 1 | | | 0 | | | | 1 | | | | 0 | | | | 0 | | | 0 | | | | 0 | | | | 1 | | | | 0 | | | | 0 | | | | 0 | | | 0 | | | | | 4 | | | | 0 | | | | | 1 | | | | 0 | | | | | 0 | | | |  |
| *Pleosporales, Pleosporaceae, Exserohilum, Exserohilum turcicum* | | 13 | | | 7 | | | | 1 | | | | 10 | | | | 3 | | | 0 | | | | 16 | | | | 8 | | | | 1 | | | | 15 | | | | 1 | | | 0 | | | | | 19 | | | | 4 | | | | | 5 | | | | 0 | | | | | 0 | | | |  |
| *Pleosporales, Pleosporaceae, NA* | | 4 | | | 0 | | | | 0 | | | | 3 | | | | 0 | | | 0 | | | | 2 | | | | 0 | | | | 1 | | | | 2 | | | | 0 | | | 0 | | | | | 0 | | | | 1 | | | | | 0 | | | | 0 | | | | | 0 | | | |  |
| *Pleosporales, Sporormiaceae, Preussia* | | 0 | | | 0 | | | | 0 | | | | 0 | | | | 0 | | | 0 | | | | 1 | | | | 0 | | | | 0 | | | | 0 | | | | 0 | | | 53 | | | | | 3 | | | | 0 | | | | | 0 | | | | 0 | | | | | 0 | | | |  |
| *Pleosporales, unidentified* | | 231 | | | 147 | | | | 10 | | | | 49 | | | | 5 | | | 12 | | | | 246 | | | | 299 | | | | 693 | | | | 219 | | | | 42 | | | 48 | | | | | 692 | | | | 71 | | | | | 59 | | | | 7 | | | | | 0 | | | |  |
| *Polyporales, Hyphodermataceae, Hyphoderma, Hyphoderma setigerum* | | 0 | | | 0 | | | | 0 | | | | 0 | | | | 0 | | | 0 | | | | 0 | | | | 0 | | | | 0 | | | | 0 | | | | 2 | | | 0 | | | | | 0 | | | | 0 | | | | | 0 | | | | 0 | | | | | 0 | | | |  |
| *Russulales, Peniophoraceae, Peniophora* | | 0 | | | 0 | | | | 0 | | | | 0 | | | | 0 | | | 0 | | | | 0 | | | | 0 | | | | 2 | | | | 0 | | | | 0 | | | 0 | | | | | 0 | | | | 0 | | | | | 0 | | | | 0 | | | | | 0 | | | |  |
| *Russulales, Stereaceae, Amylostereum, Amylostereum chailletii* | | 0 | | | 0 | | | | 0 | | | | 0 | | | | 0 | | | 0 | | | | 0 | | | | 0 | | | | 0 | | | | 0 | | | | 0 | | | 46 | | | | | 0 | | | | 0 | | | | | 0 | | | | 0 | | | | | 0 | | | |  |
| *Saccharomycetales, Debaryomycetaceae, Debaryomyces,* | | 1 | | | 1 | | | | 0 | | | | 0 | | | | 0 | | | 0 | | | | 3 | | | | 2 | | | | 2 | | | | 0 | | | | 0 | | | 103 | | | | | 0 | | | | 0 | | | | | 0 | | | | 0 | | | | | 0 | | | |  |
| *Saccharomycetales, NA* | | 22 | | | 3 | | | | 0 | | | | 2 | | | | 1 | | | 15 | | | | 3 | | | | 6 | | | | 3 | | | | 15 | | | | 2 | | | 3 | | | | | 7 | | | | 0 | | | | | 2 | | | | 0 | | | | | 1 | | | |  |
| *Saccharomycetales, Phaffomycetaceae, Wickerhamomyces, Wickerhamomyces anomalus* | | 42 | | | 2038 | | | | 2684 | | | | 2627 | | | | 2438 | | | 695 | | | | 71 | | | | 1841 | | | | 1615 | | | | 1676 | | | | 2475 | | | 819 | | | | | 16 | | | | 2561 | | | | | 2173 | | | | 2843 | | | | | 2860 | | | |  |
| *Saccharomycetales, Saccharomycetales Incertae sedis, Candida* | | 25 | | | | 2 | | | | 0 | | | | 5 | | 0 | | | | | 832 | | | | 3 | | | | 1 | | | | 4 | | | | 23 | | 39 | | | | | 398 | | | 8 | | | | | | 2 | | | | | 1 | | | | | 1 | | | | | 0 | | |
| *Saccharomycetales, Trichomonascaceae, Blastobotrys* | | 0 | | | | 0 | | | | 0 | | | | 0 | | 0 | | | | | 0 | | | | 0 | | | | 0 | | | | 0 | | | | 0 | | 0 | | | | | 2 | | | 0 | | | | | | 0 | | | | | 0 | | | | | 0 | | | | | 0 | | |
| *Saccharomycetales, Trichomonascaceae, Zygoascus, Zygoascus hellenicus* | | 0 | | | | 1 | | | | 0 | | | | 0 | | 0 | | | | | 0 | | | | 0 | | | | 0 | | | | 0 | | | | 0 | | 2 | | | | | 0 | | | 0 | | | | | | 0 | | | | | 0 | | | | | 0 | | | | | 0 | | |
| *Sporidiobolales, Sporidiobolaceae, Rhodotorula* | | 0 | | | | 0 | | | | 0 | | | | 1 | | 0 | | | | | 0 | | | | 12 | | | | 0 | | | | 2 | | | | 3 | | 0 | | | | | 0 | | | 2 | | | | | | 0 | | | | | 0 | | | | | 0 | | | | | 0 | | |
| *Sporidiobolales, Sporidiobolaceae, Rhodotorula, Rhodotorula diobovata* | | 0 | | | | 0 | | | | 0 | | | | 0 | | 0 | | | | | 0 | | | | 3 | | | | 1 | | | | 0 | | | | 0 | | 0 | | | | | 0 | | | 3 | | | | | | 0 | | | | | 0 | | | | | 0 | | | | | 0 | | |
| *Sporidiobolales, Sporidiobolaceae, Rhodotorula, Rhodotorula mucilaginosa* | | 0 | | | | 0 | | | | 0 | | | | 0 | | 0 | | | | | 94 | | | | 10 | | | | 0 | | | | 0 | | | | 0 | | 0 | | | | | 0 | | | 1 | | | | | | 0 | | | | | 1 | | | | | 0 | | | | | 0 | | |
| *Trechisporales, unidentified* | | 0 | | | | 0 | | | | 0 | | | | 0 | | 0 | | | | | 0 | | | | 0 | | | | 0 | | | | 0 | | | | 0 | | 0 | | | | | 14 | | | 0 | | | | | | 0 | | | | | 0 | | | | | 0 | | | | | 0 | | |
| *Tremellales, Cryptococcaceae, Kwoniella, Kwoniella heveanensis* | | 1 | | | | 0 | | | | 0 | | | | 0 | | 0 | | | | | 0 | | | | 4 | | | | 5 | | | | 0 | | | | 0 | | 0 | | | | | 0 | | | 0 | | | | | | 0 | | | | | 0 | | | | | 0 | | | | | 0 | | |
| *Tremellales, Cryptococcaceae, Kwoniella, Kwoniella mangrovensis* | | 1 | | | | 2 | | | | 0 | | | | 0 | | 0 | | | | | 0 | | | | 0 | | | | 4 | | | | 0 | | | | 1 | | 0 | | | | | 0 | | | 0 | | | | | | 0 | | | | | 0 | | | | | 0 | | | | | 0 | | |
| *Trichosphaeriales, Trichosphaeriaceae, Nigrospora, Nigrospora oryzae* | | | 0 | | | | 0 | | | | 0 | | | | 0 | | | 0 | | | | 0 | | | | 1 | | | | 0 | | | | 0 | | | | 1 | | | 2 | | | | 0 | | | | 0 | | | | | 0 | | | | 1 | | | | 0 | | | | | 0 | | | |
| *Wallemiales, Wallemiaceae, Wallemia, Wallemia ichthyophaga* | | | 6 | | | | 2 | | | | 0 | | | | 2 | | | 2 | | | | 0 | | | | 0 | | | | 0 | | | | 3 | | | | 5 | | | 0 | | | | 0 | | | | 1 | | | | | 0 | | | | 0 | | | | 0 | | | | | 0 | | | |
| *Xylariales, Xylariales Incertae sedis, Phialemoniopsis, Phialemoniopsis curvata* | | | 0 | | | | 0 | | | | 0 | | | | 0 | | | 0 | | | | 0 | | | | 0 | | | | 0 | | | | 0 | | | | 0 | | | 0 | | | | 0 | | | | 0 | | | | | 0 | | | | 2 | | | | 0 | | | | | 0 | | | |


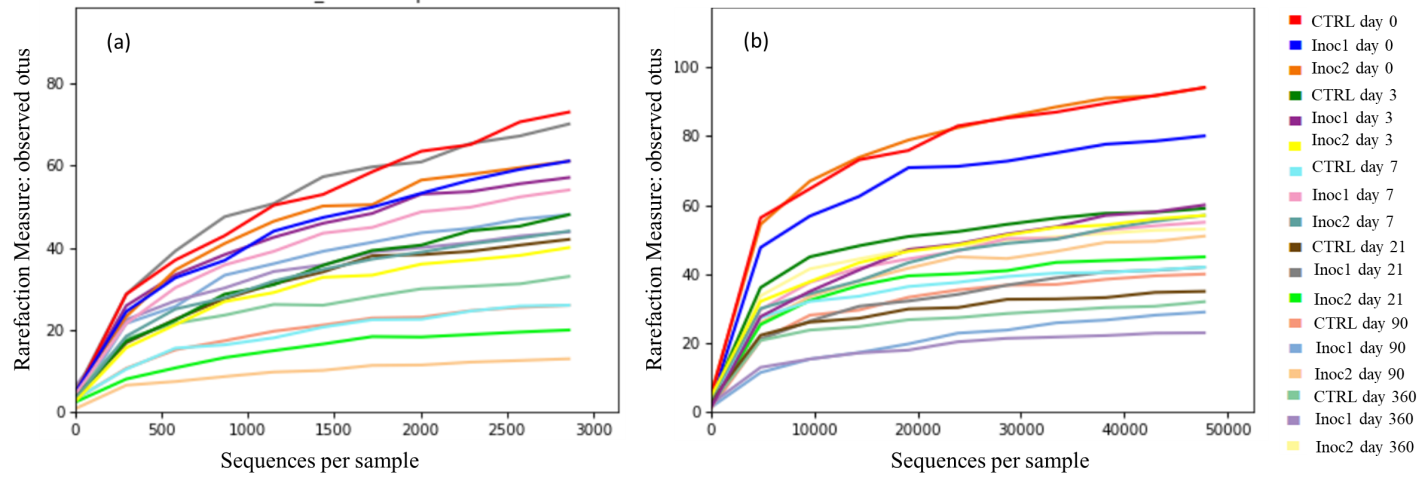


Supplementary Figure S3. Rarefaction curves showing the sampling effort and number of fungal OTUs observed in rehydrated corn (a) and sorghum (b) grain silages after 0, 3, 7, 21, 90 and 360 d of fermentation. CTRL: non-inoculated; Inoc1: *Lactobacillus plantarum* and *Propionibacterium acidipropionici* and Inoc2: *Lactobacillus buchneri.*


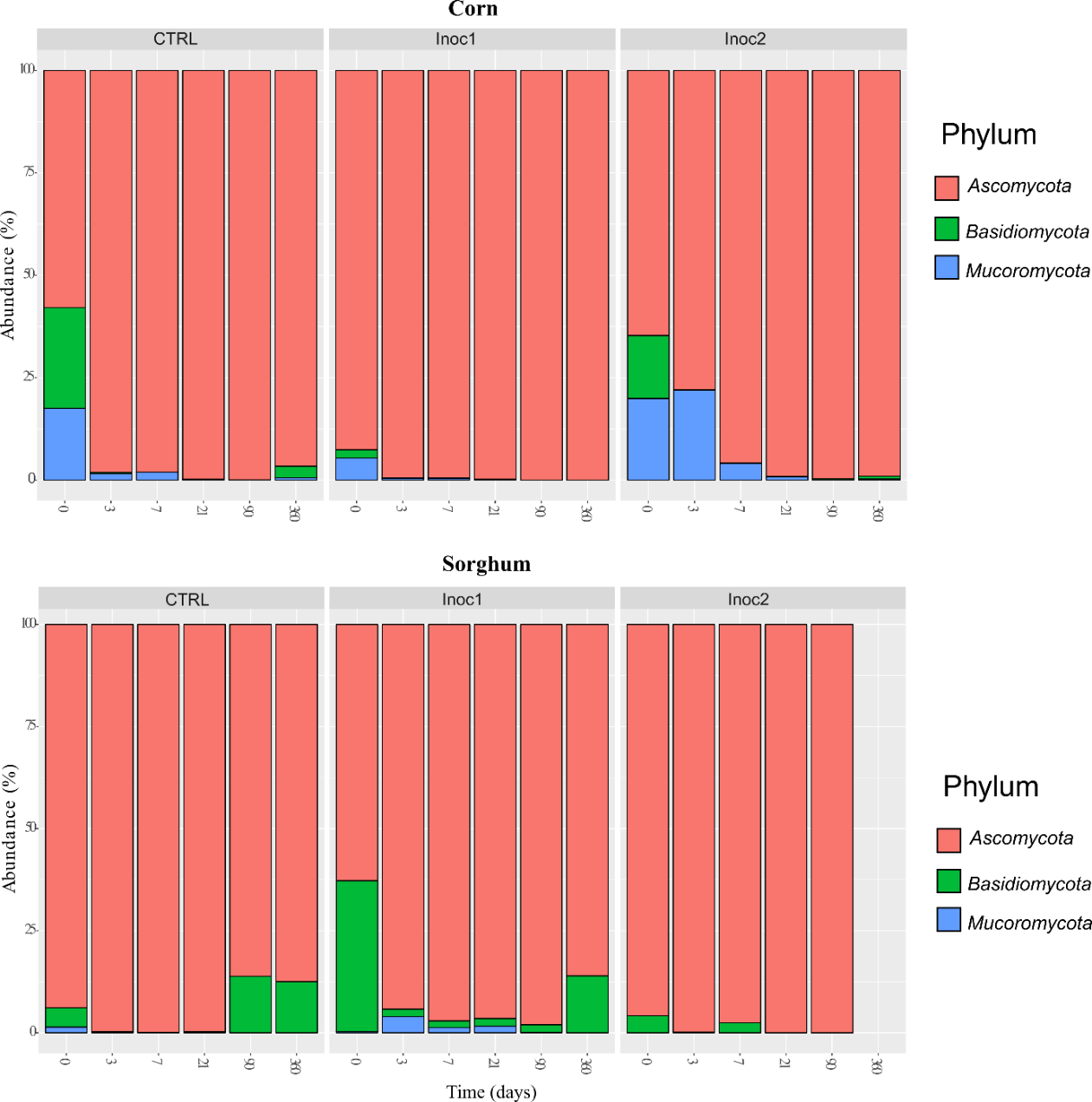


Supplementary Figure S4. Phyla taxonomic profiles of fungal communities of rehydrated corn and sorghum grain silages after 0, 3, 7, 21, 90 and 360 days of fermentation. CTRL: non-inoculated; Inoc1: *Lactobacillus plantarum* and *Propionibacterium acidipropionici* and Inoc2: *Lactobacillus buchneri*.
